# Physiotherapy scoliosis-specific exercises – a comprehensive review of seven major schools

**DOI:** 10.1186/s13013-016-0076-9

**Published:** 2016-08-04

**Authors:** Hagit Berdishevsky, Victoria Ashley Lebel, Josette Bettany-Saltikov, Manuel Rigo, Andrea Lebel, Axel Hennes, Michele Romano, Marianna Białek, Andrzej M’hango, Tony Betts, Jean Claude de Mauroy, Jacek Durmala

**Affiliations:** 1 Conservative Care for Spine and Scoliosis, ColumbiaDoctors Midtown, Columbia University Medical Center, New York, NY USA; 2 Saba University School of Medicine, Saba, Dutch Caribbean Netherlands; 3 Teesside University, Middleborough, UK; 4 Institut Elena Salvá, Barcelona, Spain; 5 Scoliosis Physiotherapy Posture and Rehabilitation Centre, Ottawa, ON Canada; 6 Asklepsios Katharina Schroth Spinal Deformities Rehabilitation Centre, Bad Sobernheim, Germany; 7 ISICO (Italian Scientific Spine Institute), Milan, Italy; 8 Italian Scoliosi Study Group (GSS), Vigevano, Italy; 9 FITS Center, Jawor, Poland; 10 Royal National Orthopaedic Hospital, London, UK; 11 Clinique du Parc, Lyon, France; 12 Department of Rehabilitation, Medical University of Silesia, Katowice, Poland

## Abstract

In recent decades, there has been a call for change among all stakeholders involved in scoliosis management. Parents of children with scoliosis have complained about the so-called “wait and see” approach that far too many doctors use when evaluating children’s scoliosis curves between 10° and 25°. Observation, Physiotherapy Scoliosis Specific Exercises (PSSE) and bracing for idiopathic scoliosis during growth are all therapeutic interventions accepted by the 2011 International Society on Scoliosis Orthopaedic and Rehabilitation Treatment (SOSORT). The standard features of these interventions are: 1) 3-dimension self-correction; 2) Training activities of daily living (ADL); and 3) Stabilization of the corrected posture. PSSE is part of a scoliosis care model that includes scoliosis specific education, scoliosis specific physical therapy exercises, observation or surveillance, psychological support and intervention, bracing and surgery. The model is oriented to the patient. Diagnosis and patient evaluation is essential in this model looking at a patient-oriented decision according to clinical experience, scientific evidence and patient’s preference. Thus, specific exercises are not considered as an alternative to bracing or surgery but as a therapeutic intervention, which can be used alone or in combination with bracing or surgery according to individual indication. In the PSSE model it is recommended that the physical therapist work as part of a multidisciplinary team including the orthopeadic doctor, the orthotist, and the mental health care provider - all are according to the SOSORT guidelines and Scoliosis Research Society (SRS) philosophy. From clinical experiences, PSSE can temporarily stabilize progressive scoliosis curves during the secondary period of progression, more than a year after passing the peak of growth. In non-progressive scoliosis, the regular practice of PSSE could produce a temporary and significant reduction of the Cobb angle. PSSE can also produce benefits in subjects with scoliosis other than reducing the Cobb angle, like improving back asymmetry, based on 3D self-correction and stabilization of a stable 3D corrected posture, as well as the secondary muscle imbalance and related pain. In more severe cases of thoracic scoliosis, it can also improve breathing function.

This paper will discuss in detail seven major scoliosis schools and their approaches to PSSE, including their bracing techniques and scientific evidence. The aim of this paper is to understand and learn about the different international treatment methods so that physical therapists can incorporate the best from each into their own practices, and in that way attempt to improve the conservative management of patients with idiopathic scoliosis. These schools are presented in the historical order in which they were developed. They include the Lyon approach from France, the Katharina Schroth Asklepios approach from Germany, the Scientific Exercise Approach to Scoliosis (SEAS) from Italy, the Barcelona Scoliosis Physical Therapy School approach (BSPTS) from Spain, the Dobomed approach from Poland, the Side Shift approach from the United Kingdom, and the Functional Individual Therapy of Scoliosis approach (FITS) from Poland.

## Background

In recent decades, there has been a call for change among all stakeholders involved in scoliosis management. Parents of children with scoliosis have complained about the so-called “wait and see” approach that far too many doctors use when evaluating children’s scoliosis curves between 10° and 25° [1]. Numerous physical therapists have reported that children with scoliosis and their parents are reacting to their lack of empowerment as they wonder how to help themselves beyond simply waiting and bracing. Physical therapists, most of whom are still inadequately educated and equipped to provide quality scoliosis treatment, have searched for new treatment methods. Orthotists have recognized that traditional braces lack the ability to make 3D corrections, producing flat back or other poor cosmetic changes, and are looking for more effective options. Finally, doctors have sought out alternatives to help patients who are not good candidates for surgery [2].

The Society of Scoliosis Orthopedic Rehabilitation and Treatment (SOSORT) was founded in 2004 in reaction to this growing awareness. SOSORT promotes and encourages conservative, evidence-based medicine regarding scoliosis and provides education, guidelines and consensus about treatment options to people with scoliosis [3]. Every scoliosis approach or ‘school’ around the world subscribes to SOSORT’s principles and shares a common mission. The shared goal is not simply to look at the spine in the coronal plane but to look at the affected subject and family under a more holistic psychosocial model, where present and future quality of life is the main objective.

SOSORT uses the term Physiotherapy Scoliosis Specific Exercises (PSSE) in connection with all of the schools represented within the organization. The effectiveness of PSSE in treating patients with Adolescent Idiopathic Scoliosis (AIS) has been demonstrated by recent studies. While a Cochrane review published in 2012 [4] reported a low to very low quality of evidence for the proposition that PSSE was effective in improving Cobb angle, angle of trunk rotation, pain and quality of life, since the time of that review, four randomized controlled trials (RCTs), which are generally recognized as the highest level of evidence for primary studies, have provided strong proof that PSSE are indeed effective for treating AIS patients with mild and moderate curves. The four RCTs were conducted in different parts of the world – in Italy by Monticone et al. [5] (2013), in Canada by Schreiber et al. [1] (2015), in England by Williamson et al. 2015 [6], and in Turkey by Kuru et al. [7] (2015) – and are summarized in the body of this paper.

Seven major scoliosis schools and their approaches to PSSE, including their bracing techniques, will be discussed in detail in this paper. The differences between the schools are related to PSSE used by each school. The purpose of this paper is not to determine which scoliosis school and treatment approach is superior to the others. Rather, the aim is to understand and learn about the different treatment methods around the world so that physical therapists can incorporate the best from each into their own practices, and in that way attempt to improve the conservative management of patients with idiopathic scoliosis.

The schools are presented in the historical order in which they were developed. They include the Lyon approach from France, the Katharina Schroth Asklepios approach from Germany, the Scientific Exercise Approach to Scoliosis (SEAS) from Italy, the Barcelona Scoliosis Physical Therapy School approach (BSPTS) from Spain, the Dobomed approach from Poland, the Side Shift approach from the United Kingdom, and the Functional Individual Therapy of Scoliosis approach (FITS) from Poland.

## The Lyon approach (France)

### Introduction

**Fig. 1:**
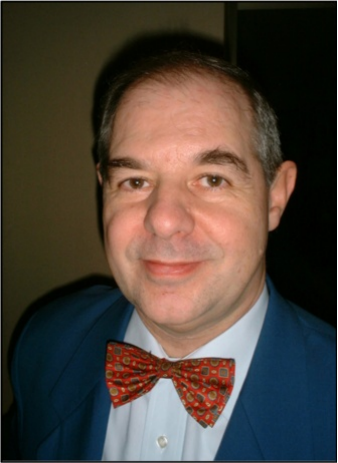
Dr. Jean Claude de Mauroy, co-inventor of the new Lyon ARTbrace (Asymmetrical Rigid Torsion Brace)

The Lyon school of physiotherapy for scoliosis, managed by Dr. Jean Claude de Mauroy, the head of the orthopedic medicine department at Clinique du Parc, Lyon, France (Fig. 1), is one of the oldest physiotherapy schools in France and one of the first schools to be integrated into the Faculty of Medicine program in Lyon. Physiotherapy is an integral part of the Lyon approach to the management of scoliosis in conjunction with casting and bracing.

### History

Dr. Gabriel Pravaz, an orthopedic surgeon, created the first orthopedic physiotherapy center in Lyon two centuries ago. In the middle of the 20th century, Dr. Pierre Stagnara established an organized nonsurgical approach to manage scoliosis with casts and braces, and in 1947 he created the Lyon brace. More recently, the ARTbrace has been developed, which obviates the need for a plaster cast [8]. Although the Lyon method is primarily focused on the use of bracing – and in the recent past on both casting and bracing – it includes scoliosis specific exercises to support treatment.

### Definition of treatment

The Lyon method traditionally combined PSSE with the Lyon brace and casting, and more recently has combined PSSE with bracing alone in the form of the new Lyon ARTbrace (Asymmetrical Rigid Torsion brace). Physiotherapeutic treatment includes 3D mobilization of the spine, mobilization of the ilio-lumbar angle (lumbar scoliosis), patient education, and activities of daily living, including correction of the sitting position.

### Treatment indications, goals and age specifics

The 2011 SOSORT Guidelines provide clear, scientific indications regarding what type of treatment (observation, physical therapy, bracing, surgery) is appropriate for patients with scoliosis [9]. Under the Lyon approach, the treatment is more specifically determined by the type of scoliosis; chaotic or linear [10]. Chaotic scoliosis is a true 3D structural deformity of the spine, which occurs in approximately 2.5 % of adolescents with scoliosis curves <20° Cobb angle. This is a dynamic scoliosis, which can be influenced by many environmental factors. Because of the uncertainty of its progression, chaotic scoliosis can best be described by deterministic chaos. While, according to Newton’s Law of Gravity, we can predict where the apple that falls from a tree will land, we cannot similarly predict where a leaf that falls from a tree will land. The tree leaf falls according to the same laws, but the precise location of its landing remains unpredictable because the leaf is more sensitive to the wind. This type of unpredictability also defines the development and progression of scoliosis in an individual. The spine is highly sensitive to the influence of the nervous system on its growth and development, and any change in the nervous system leads to the deterministic chaos of scoliosis below 20°. The progression of scoliosis is neither certain nor predictable as the nervous system is constantly trying to adapt and correct the asymmetric growth during the early development of the scoliosis [11].

**Fig. 2:**
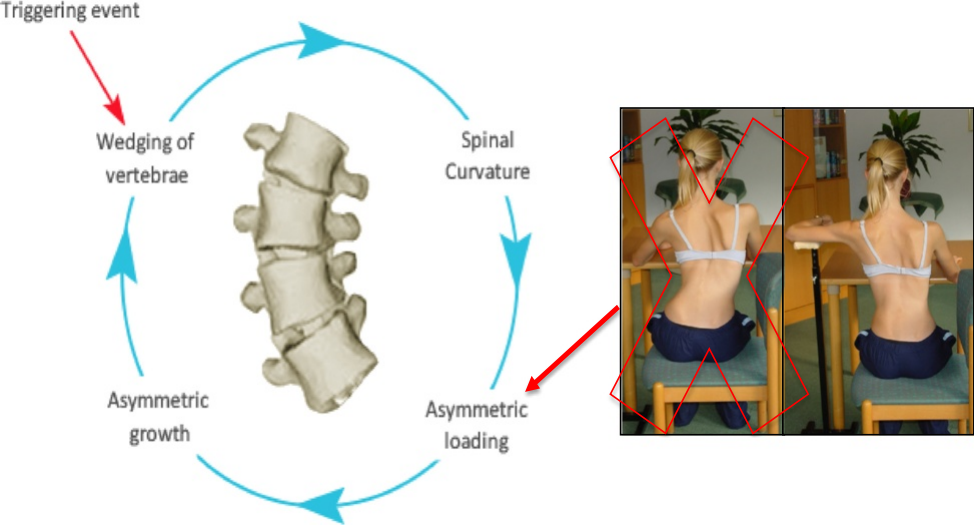
Image on the left: The Vicious cycle. Dr. Stokes and Burwell hypothesized that the vicious cycle of scoliosis curve progression begins with a triggering event which leads to the formation of wedged vertebrae. Wedged vertebrae cause the spine to curve which results in continuous asymmetric loading on the spine. This in turn, can potentially promote asymmetric growth of the spine and progression of scoliosis curves as asymmetric growth increases the wedging of the vertebrae and perpetuates the cycle to continue. The image on the right shows a scoliosis patient sitting with increased asymmetric loading of the spine as described in Stokes’ ‘Vicious Cycle”. The large red “X” on the image indicates that this is not the desired posture. The image on the right shows the same scoliosis patient sitting with improved asymmetric loading of the spine as she performs scoliosis specific physiotherapy exercises in accordance with the Lyon approach

The other category of scoliosis is linear scoliosis, which occurs in approximately 0.25 % of adolescents with scoliosis curves >20°. Madame Duval Beaupère initially described the linear progression of scoliosis in polio patients. A triggering event brings scoliosis into a ‘vicious cycle’ (Fig. 2) that was later described by Ian Stokes and R. Geoffrey Burwell in detail in their study on the biomechanics of scoliosis progression [12]. Stokes and Burwell explain that the vicious cycle begins with a triggering event, which results in the formation of wedged vertebrae. What follows is continuous asymmetric loading on the spine, which can potentially promote asymmetric growth and advancement of the progression. The aim of PSSE is to intervene in this vicious cycle at the level of patient education and promotion of spinal and trunk alignment by reducing or even stopping the asymmetric loading and by potentially helping to stop scoliosis progression.

The goals of the Lyon method are improved motivation with bracing, patient education, including awareness of postural defects, and increased range of motion, neuromuscular control of the spine, coordination, trunk stabilization, muscular strength, respiration, and ergonomics.

The scoliosis treatment protocol of the Lyon method depends on the patient’s age. Juvenile patients (younger than 15 to 17 years) do not do stretching. Adolescent patients complete the entire program. With adult patients, the focus is on pain reduction and disc protection.

### Classification system

The classification system used for physiotherapy and for bracing are the Ponseti and the Lenke classifications, respectively.

### Principles of the Lyon method

The Lyon method of scoliosis treatment involves five stages:

1. The Lyon approach to assessment.
2. Awareness of trunk deformity.
3. What to do: sample exercises.
4. What not to do and why.
5. Sport or only physiotherapy?

#### Stage I: Lyon approach to assessment

The Lyon approach considers three factors in determining the regimen of therapy to pursue: the patient’s age, postural imbalance, and the Cobb angle.

#### Stage II: Awareness of trunk deformity

**Fig. 3:**
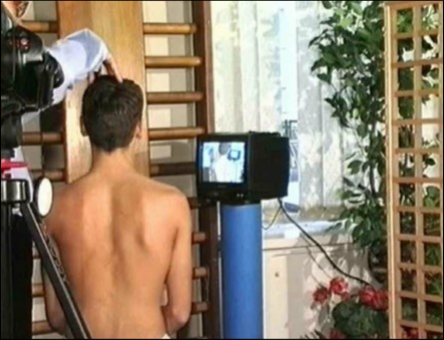
Scoliosis patient developing self-awareness of postural defects with the help of a video recorder and real-time video feedback

The Lyon approach uses visualization with mirrors and video to help with curve correction (Fig. 3).

#### Stage III: What to do: sample exercise

**Fig. 4:**
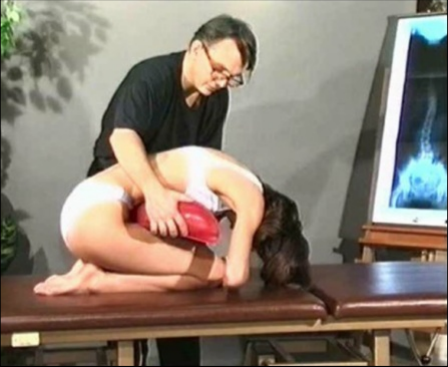
Active thoracic mobilization, promoting kyphosis, using the Lyon method

**Fig. 5:**
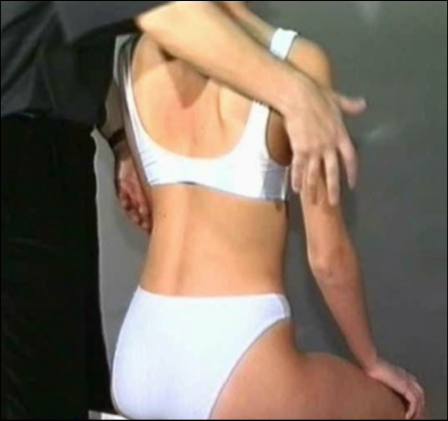
Active lumbar correction, promoting lordosis, using the Lyon method

**Fig. 6:**
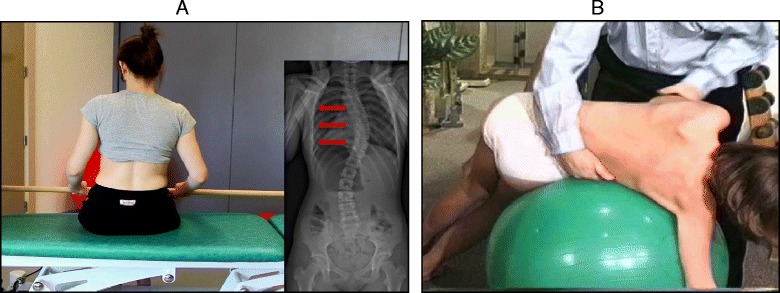
(**a**, **b**): Active thoracic shift exercise with a dowel (**a**) and a Swiss-ball (**b**) using the Lyon method

**Fig. 7:**
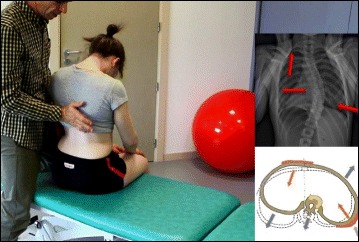
Active thoracic shift and derotation exercise using the Lyon method. *Arrows* in the radiograph and the diagram show the direction of the thoracic shift and the derotation of the ribcage as the exercise is performed using the Lyon method

**Fig. 8:**
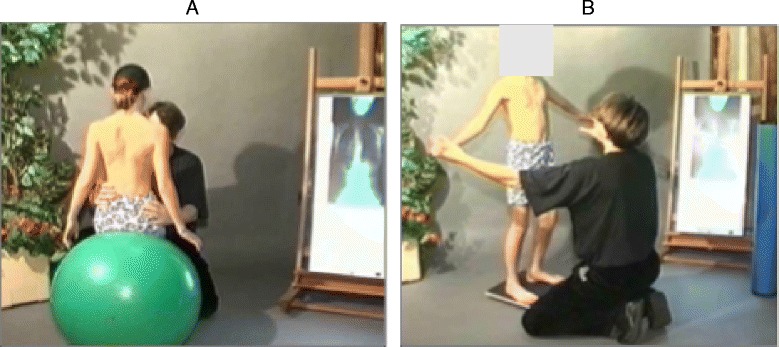
(**a**, **b**): Balance and proprioception exercises on a Swiss-ball (**a**) and on a balance board (**b**) using the Lyon method

**Fig. 9:**
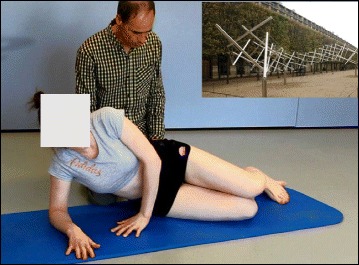
Spinal stabilization exercises using the Lyon method

**Fig. 10:**
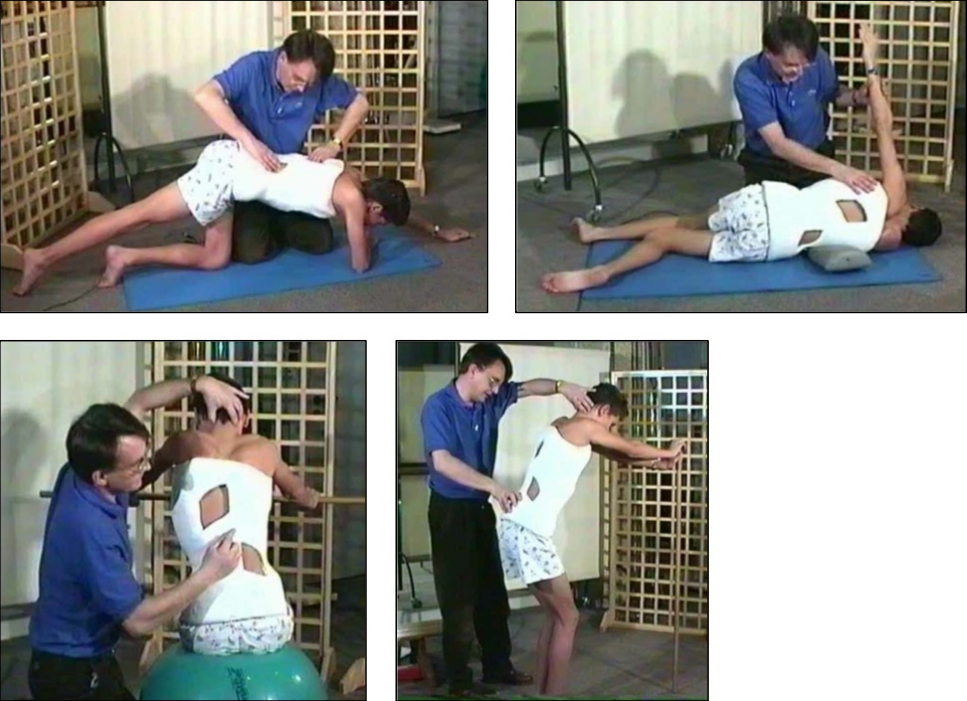
Several standard Lyon exercises in a Lyon plaster cast promoting core strength (top left), breathing and thoracic shift (bottom), and elongation

**Fig. 11:**
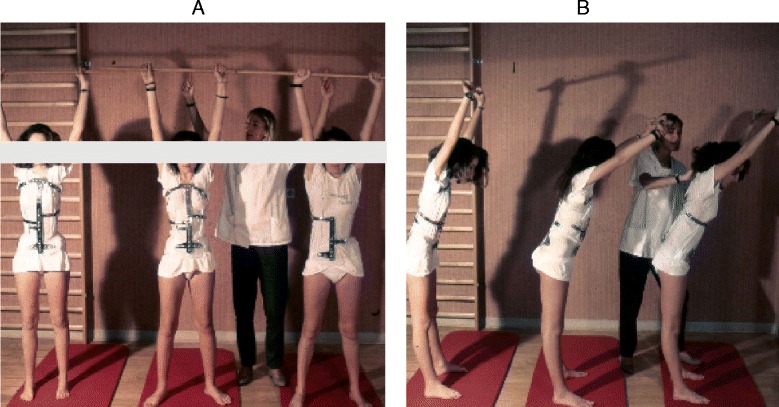
(**a**, **b**): Several standard Lyon exercises in a Lyon plaster cast promoting postural correction (**a**) and core strengthening (**b**)

The basis of the Lyon method is to avoid spinal extension during exercise and enhance kyphosis of the thoracic region with lordosis of the lumbar spine as well as frontal plane correction, segmental mobilization, core stabilization, proprioception, balance and stabilization (Figs. 4, 5, 6, 7, 8 and 9). In the Lyon approach, a great emphasis is given to exercises done in the plaster cast prior to bracing (Fig. 10) and during bracing (Fig. 11) to encourage equilibrium and muscular strength and endurance while in the cast or brace.

#### Stage IV: What not to do and why

The Lyon method avoids sagittal plane extreme movements (flexion and extension) and exercises causing shortness of breath.

#### Stage V: Sport or only physiotherapy?

**Fig. 12:**
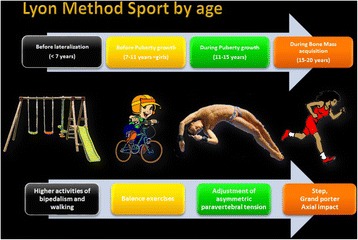
Activity level recommendations by age per the Lyon School in accordance with the Lyon treatment principles

The Lyon method teaches patients how to play sports and the best and worst sports for scoliosis (Fig. 12).

### The use of breathing mechanics, muscle activation, and mobilization

**Fig. 13:**
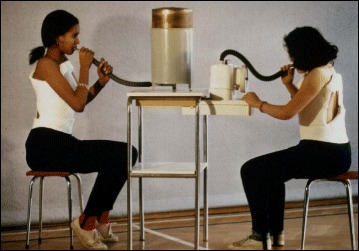
Lyon method breathing exercises using a breathing machine, performed while wearing a Lyon plaster cast, increases lung capacity

**Fig. 14:**
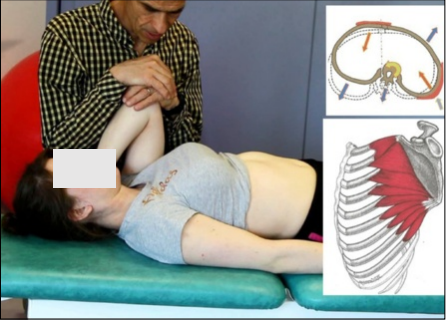
Active thoracic mobilization using the Lyon method. *Arrows* in the diagram on the right show the direction of thoracic mobilization of the ribcage

**Fig. 15:**
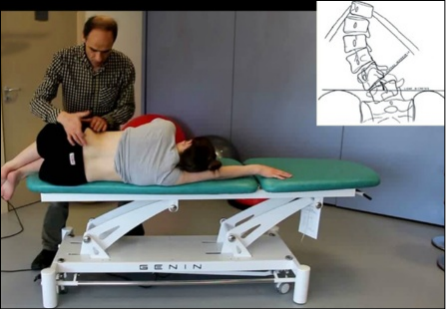
Active lumbar mobilization using the Lyon method. The diagram on the right shows lumbar scoliosis

**Fig. 16:**
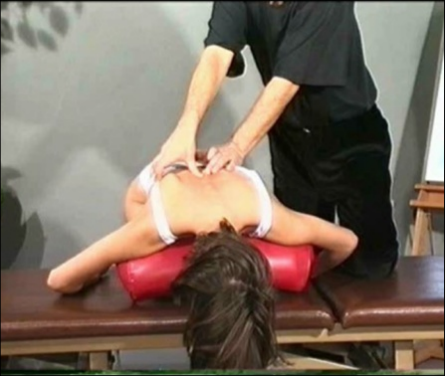
Mobilization of the costovertebral joints using the Lyon method

The Lyon method uses rotational angular breathing with the diaphragm as well as a breathing machine to increase lung capacity (Fig. 13). The Lyon method also improves the endurance of the deep paraspinal and core musculature and focuses on mobilization to improve correction (Figs. 14, 15 and 16).

### Treatment tools: active and passive

The Lyon method uses mirrors and video to assist in correction and to help patients develop perception of their spine and their postural defects.

### Description of the most relevant exercise mechanics (see Figures in section Classification system)

1. Lying: kyphotization with a cushion.
2. Rolling: fetal position with a cushion and derotation on a Swiss ball in kyphosis.
3. Sitting: adjustments of the lumbar lordosis in the sitting position and mobilization on a Swiss ball.
4. Standing.

### Activities of daily living and sport

**Fig. 17:**
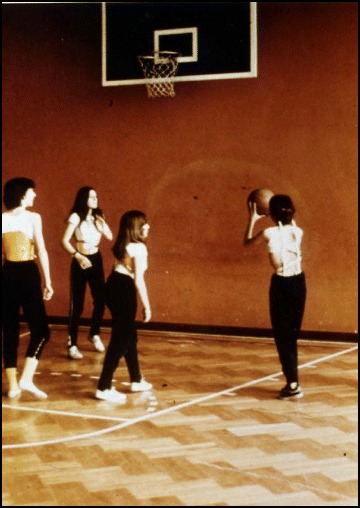
The Lyon method encourages athletic activities while wearing the Lyon brace. This group of scoliosis patients are playing basketball, which aids vertical stretching and spinal flexibility

The Lyon method helps patients develop the correct posture while sitting at a table to write or type on a computer. Sports, such as basketball (Fig. 17), are an essential part of the Lyon method.

### Scientific evidence

The Lyon approach is not supported by scientific evidence for cases where the Cobb angle is less than 20°. For cases where the Cobb angle is 20° or greater, the method depends primarily on casting and bracing for its effectiveness. Under this approach, the physical therapy exercises are properly viewed as supplemental to the casting and bracing, and in each case are adapted to the individual’s particular needs. As expressed by Dr. Jean Claude de Mauroy, the physical therapy elements of the Lyon approach are better described as the “Lyon experience” than the “Lyon method” [10]. It must be noted that, while outside the scope of this paper, there does exist scientific support for the effectiveness of the casting and bracing espoused by the Lyon approach.

### The ARTbrace

**Fig. 18:**
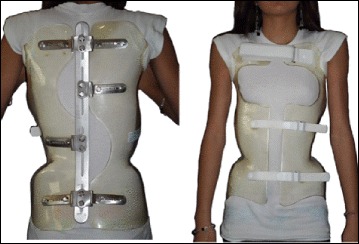
Anterior (*left*) and posterior (*right*) views of the new asymmetric rigid torsion brace (ARTbrace) made from 4 mm polycarbonate. The main biomechanical concepts are based on elongation along the vertical axis, lateral inflexion in the frontal plane, and derotation of the spine in order to obtain a correction of the scoliotic curve

The ARTbrace (Fig. 18) is a new brace: asymmetric, rigid, made from 4 mm polycarbonate [8]. The brace reproduces the shape of a twisted column opposite to the scoliosis. Both polycarbonate lateral hemi-shells are articulated on a posterior metal bar. Both anterior and inferior closures are rigid; the upper third is a velcro strap. The ARTbrace is the only asymmetrical brace with lateral hemi-shells.

As with the old Lyon brace, the ARTbrace is adjustable, but many new concepts were used to build the brace:

1. The mathematical basis of the detorsion is the circled helicoid with horizontal circle generator.
2. The multiple 3 points system is replaced by a global detorsion.
3. Three regional 2D individual moldings are superposed to obtain a 3D helicoidal correction with coupled movements.
4. The sagittal plane is fixed in a physiological posture to improve flat back if necessary.
5. The upper part of the brace supports the trunk like a baby lift.
6. In the middle, under the breast, the clamping of the two hemi-shells realizes the “tube mayonnaise” effect with passive axial lengthening and geometric detorsion.
7. The polycarbonate-skin interface is a soft contact type with mechanical detorsion of a cylinder.
8. Global detorsion is like a wrench and bolt along the vertical axis.

The Lyon method of physiotherapy is adapted to prepare the child in order to facilitate the regional molding and detorsion over a period of time.

**Fig. 19:**
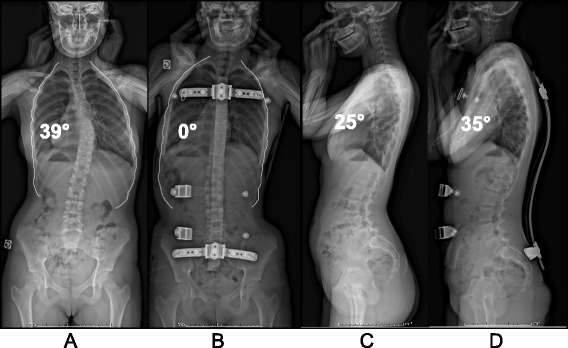
(**a**, **b**, **c**, **d**): Radiographic series of a female patient with progressive scoliosis (patient ‘S’). Initial PA (**a**) and lateral (**c**) radiographs at the time of diagnosis at age 13 show a thoracic T5-T12 scoliosis of 39° Cobb angle (**a**) and a 25° hypokyphosis (**c**). Repeat radiographs taken in the ARTbrace show a decreased Cobb angle on PA radiograph (**b**) and an increased kyphosis on lateral radiograph (**d**)

**Fig. 20:**
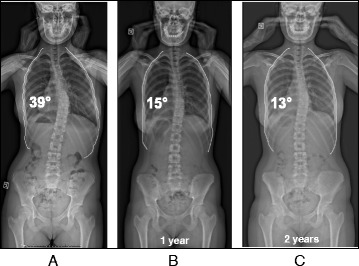
(**a**, **b**, **c**): Radiographic series of patient ‘S’ at one year (**b**) and two years (**c**) after brace weaning. Compared to the initial radiograph (**a**), the final radiograph (**c**) shows a reduction in curve Cobb angle of over 50 %

In Figs. 19 and 20 the case of ‘S’ demonstrates the results of brace wearing over two years in a progressive case.

## The Schroth method (Germany)

### Introduction

**Fig. 21:**
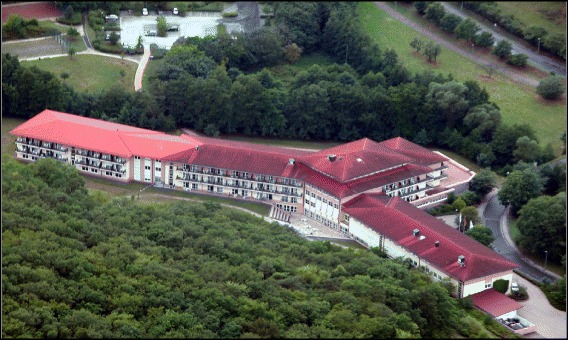
Asklepios Katharina Schroth Spinal Deformities Rehabilitation Centre in Bad Sobernheim, Germany. Formerly called the Katharina Schroth Klinik

Based upon typical physiotherapeutic principles, the Schroth method was developed by Katharina Schroth in 1920, and continuously refined through the treatment of approximately 3,000 scoliosis cases per year. The Asklepios Katharina Schroth Spinal Deformities Rehabilitation Centre in Germany (Fig. 21) offers a scoliosis-specific intensive inpatient rehabilitation program. In addition to the treatment offered at the Centre, 2,500 trained and certified Schroth therapists treat patients through the center’s residential outpatient treatment program.

**Fig. 22:**
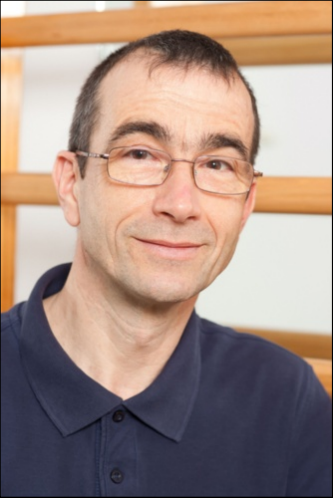
Axel Hennes, head of the physical therapy department at the Asklepios Katharina Schroth Spinal Deformities Rehabilitation Centre in Bad Sobernheim, Germany

The broad network of therapists enables the continuation and actualization of the Schroth method throughout much of the world, including in Germany, Russia and many other European countries, in Canada and the United States, in Australia, and several countries in Asia. The leading educator of Schroth therapists is Axel Hennes (Fig. 22), who is the head physical therapist at the Medical Spine Center in Bad Sobernheim, Germany. Another central figure in the school today is Dr. Hans Weiss, the grandson of Katharina Schroth, who has published numerous studies regarding the Schroth method (see details in Scientific evidence).

The main goals of the Schroth method are to provide effective treatment for patients, and training and education for physiotherapists. The treatment approach includes both intensive inpatient rehabilitation and residential outpatient physiotherapy provided by certified Schroth therapists [13].

### History

**Fig. 23:**
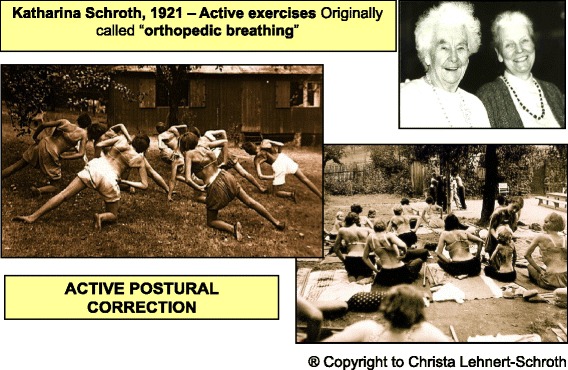
History of the Schroth method. Katharina Schroth with her daughter, Christa Lehnert-Schroth (*top right*). Patients with scoliosis exercising outdoors at the Katharina Schroth Klinik (*bottom right*; *left*)

Katharina Schroth, born in Dresden, Germany, in 1894, was suffering from moderate scoliosis and underwent treatment with a steel brace before she decided to develop a more functional approach to treat her scoliosis and improve her quality of life. Inspired by the way in which a balloon is inflated, in 1910 she tried to correct her own deformity by breathing into the concavities of her trunk in front of a mirror. She recognized that 3D postural correction could only be achieved with a series of corrective exercises designed to support a corrected posture and change the postural perception of the person suffering with scoliosis. The principles of active 3D posture correction, corrective breathing, and correction of postural perception form the foundation for what came to be known as the Schroth method of scoliosis treatment [14] (Fig. 23).

By 1921, Katharina Schroth’s success with her own scoliosis was attracting attention, and with the help of her daughter, Christa Lehnert-Schroth, she began treating others with scoliosis in her small institute in Meissen, Germany. By the late 1930’s, the Schroth method was widely recognized as the best conservative scoliosis treatment method in all of Germany. After World War II, Katharina Schroth and her daughter moved to West Germany and opened an institute in Bad Sobernheim, which soon grew into a full-scale scoliosis treatment clinic that served more than 150 inpatients at a time [14]. In the 1980’s, the institute was renamed the Asklepios Katharina Schroth Klinik.

Hans-Rudolf Weiss, an orthopedic surgeon, and the grandson of Katharina Schroth, was the medical director of the Asklepios Katharina Schroth Rehabilitation Centre from 1995 to 2008. In the summer of 2009 he opened his own practice for orthopedics and rehabilitation and now offers new concepts of bracing and physiotherapy based on the Schroth method. He has conducted and published a substantial amount of research supporting the effectiveness of the Schroth method on improving the Cobb angle, the angle of trunk rotation, vital capacity, pain, quality of life, as well as the effectiveness of bracing in reducing the need for surgery. (Details of these studies can be found in Scientific evidence).

Today, the Asklepios Katharina Schroth Klinik accommodates 200 inpatients and has a waiting list for its renowned treatment courses. Christa Lehnert-Schroth (1924–2015) was involved in the treatment of more than 10,000 scoliosis patients over the course of her 50-year career at the Asklepios Schroth Klinik.

### Classification system

**Fig. 24:**
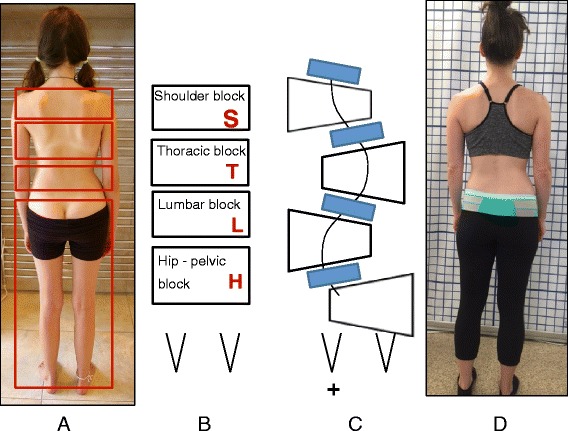
(**a**, **b**, **c**, **d**): Schroth Body Blocks. The Schroth system of scoliosis curve classification is derived from the Schroth principle of dividing the body into “body blocks” as pictured anatomically (**a**) and schematically (**b**). Scoliosis causes the body blocks to become deformed, changing their geometric shape from a rectangle (**b**) to a trapezium (**c**). A patient with a major lumbar scoliosis left convex curve has a lumbar block shifted to the left and a hip-pelvic block shifted to the right (**d**)

The Schroth system of classification [14] is derived from the Schroth principle of dividing the body into ‘Body Blocks’. This symbolic description helps to explain the scoliotic alterations as compensatory adaptations. The Body Blocks depict the trunk deformation as a change in their geometric form from a rectangle to a trapezium shape. Side-shift and rotation as well as compression on the concave side and expansion on the convex side are clearly visible. In the standing static position the body blocks should be aligned perpendicularly with their center of gravity integrated in the central sacral line (CSL) as seen in Fig. 24. The scoliotic trunk asymmetry is a loss of symmetry and shows the blocks skewed and off-center (Fig. 24).

The Schroth classification system gives the direction of the side deviation and rotation of the main important body blocks (major curves) and a clear orientation for the standardized therapy plan which includes the therapy diagram, exercise-program with home-exercises, and necessary mobilizing technique.

According to the Schroth classification system, the different scoliosis types always start with the major curve and are followed by relevant secondary curves.

The uppercase letters represent the body blocks and the lowercase letters describe the direction of the lateral deviation and rotation: right = ri, left = le. Schroth body blocks:

**H** – Hip-pelvic block including the lower limbs reaching the lower end vertebra (LEV) of the lumbar curve.

**L** – Lumbar block enclosed by upper end vertebra (UEV) and LEV of the lumbar curve or thoracolumbar curve respectively.

**T** – Thoracic block between UEV and LEV of the thoracic curve.

**S** – Shoulder block represents the cervical thoracic (proximal thoracic) curve located between UEV of the thoracic curve and UEV of the proximal thoracic curve.

The following is an overview of the classifications:

1. Thoracic scoliosis (means that the major curve is located in the thoracic spine, and the curve can be to the right or to the left).
  - Thoracic only.
  - Thoracic with lumbar to opposite side with hips in center.
  - Thoracic with lumbar and hips protruding to the opposite side of the thoracic curve (along with the lumbar).
2. Lumbar scoliosis (means that the major curve is located in the lumbar spine, and the curve can be to the right or to the left).
  - Lumbar only with hips protruding to the opposite side of the curve.
  - Lumbar curve with thoracic and hips protruding to the opposite side of the lumbar curve.
  - Lumbar and thoracic curves with hips in center.
3. Sagittal plane deformities including increased thoracic kyphosis (round back), decreased thoracic kyphosis (flat back) and increased lumbar kyphosis or loss of the normal anatomical lordosis (curve) of the lumbar spine.

### Treatment indications and goals

Treatment indication for the Schroth method is based on the SOSORT guidelines [9].

Both individual and group treatments share these same goals:

1. Proactive spinal corrections to avoid surgery.
2. Postural training to avoid or decelerate progression.
3. Information to support a decision-making process.
4. Teaching a home-exercise program.
5. Support help for self-help.
6. Prevention and coping strategies for pain.

### Age specifics

The Schroth method is primarily used for idiopathic scoliosis, including AIS and late juvenile idiopathic scoliosis (JIS). People with early onset scoliosis (EOS), and adults, are treated with modified principles. Sagittal plane deformities such as hyper-kyphosis (Scheuermann’s kyphosis) and lordosis (inverted back) can also be treated with Schroth exercises. Treatment of JIS involves a less intense and modified Schroth method as well. Treatment of AIS using strict Schroth principles is aimed at preventing curve progression before the end of growth. Treatment of adult onset scoliosis implements a modified Schroth method based on the severity of pain and the degree and rigidity of the spinal deformity.

### 3D principles of correction

In the Schroth method there are five pelvic corrections that are assumed prior to the execution of the main principles of correction. These five pelvic corrections ensure that the pelvis is best aligned with the trunk prior to the major corrections.

The five principles of the Schroth method are: 1) Auto-elongation (detorsion); 2) Deflection; 3) Derotation; 4) Rotational breathing; and 5) Stabilization. During the application of these principals, as with the BSPTS method, the patient is taught how to de-collapse the concaved areas of the trunk and how to reduce the prominences.

### The use of breathing mechanics, muscle activation, and mobilization

The use of specific and special Rotation Angular Breathing (RAB) (also called orthopaedic breathing) will be discussed in detail in The Use of Breathing Mechanics, Muscle Activation, and Mobilization below.

The method also includes mobilization and flexibility in the spine and between ribs to enhance joint mobility prior to the exercises. Muscle activation is done via specific activation of muscles that can improve the correction, such as the iliopsoas, the quadratus lumborum and erector spinae.

**Fig. 25:**
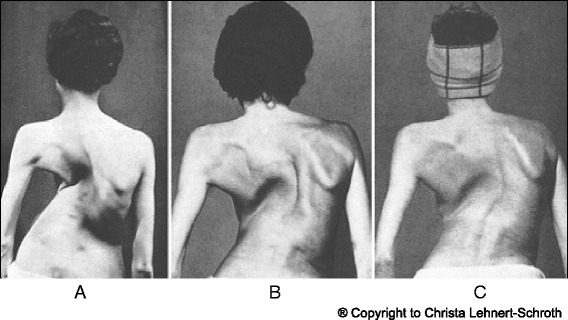
(**a**, **b**, **c**): Severe scoliosis in a 24-year-old female patient. Initial photograph (**a**) before beginning scoliosis treatment shows total left trunk atrophy with a prominent right thoracic rib hump. Photographs of the same patient 9 months (**b**) and 12 months (**c**) after intensive scoliosis therapy with rotational angular breathing (RAB) exercises (also called orthopaedic breathing exercises) according to the Schroth method show a visible improvement of the scoliosis

**Fig. 26:**
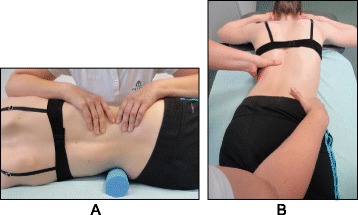
Schroth method lumbar mobilization (**a**) and curve flexibility (**b**) exercises

In Figs. 25 and 26 the use of RAB and specific mobilization and flexibility are demonstrated.

### Description of Schroth method exercises

**Fig. 27:**
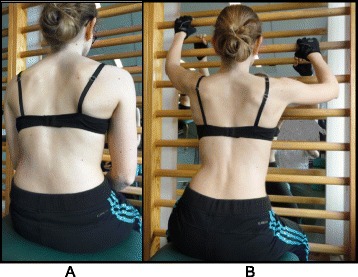
The Schroth “50 x Pezziball” exercise where the patient sits on a Swiss-ball in front of a mirror (**a**) and performs active 3D auto self-correction using the wall bar (**b**)

Four of the most commonly used exercises in the Schroth method are the “50 x Pezziball” exercise, Prone exercise, Sail exercise, and the Muscle-cylinder exercise. All of these exercises can be used for all curve types. The “50 x Pezziball” exercise works on auto-self-elongation and activation of muscles in the trunk that force the convexities in the trunk “forward and inward” and the concavities “outward and backward” (Fig. 27).

**Fig. 28:**
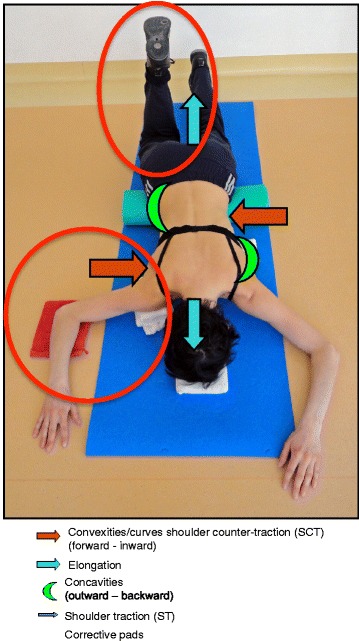
The Schroth prone exercise with activation of the iliopsoas muscle (right hip flexion). *Blue arrows* represent trunk elongation with caudal and cranial forces. *Red arrows* represent areas of muscle activation around the convexities towards the midline. *Green half-moons* represent areas of expansion of the concavities. *Red circles* represent additional corrective forces: *red circles* around the right lower extremity and the right upper extremity represent iliopsoas activation and shoulder traction/counter-traction, respectively, resulting in correction of the lumbar and thoracic curves

**Fig. 29:**
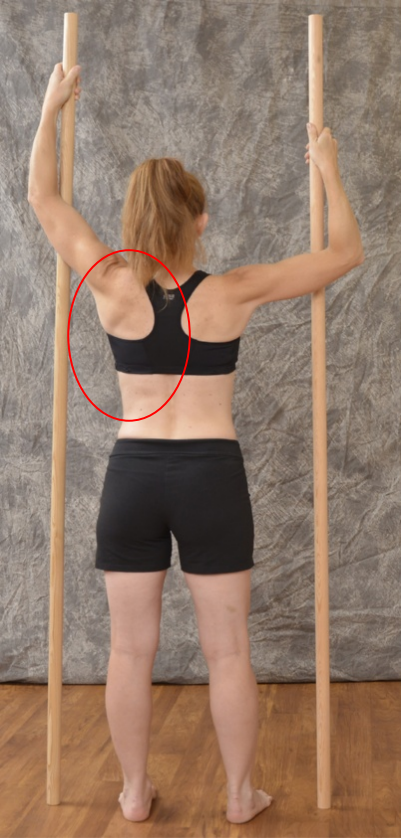
The Schroth “Sail” exercise where the patient stands on a half foam-roll with two poles and performs active stabilization. The red circle represents the concavity (weak side according to Schroth). During active stabilization, the patient is consciously expanding the *left rib cage* with right directional breathing, opening the collapsed *left lung*, while maintaining 3D postural correction

**Fig. 30:**
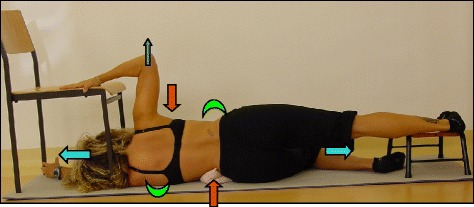
The “Muscle-cylinder” exercise (also known as the “Side-lying” exercise), focusing mainly on the correction of the lumbar scoliosis curve. During this exercise, the patient lies on the lumbar convex side. The lumbar convexity is supported by a rice bag to help align the spine in the horizontal plane. The patient’s right leg is supported by a stool (in case of 4C/major lumbar scoliosis) and the patient’s right arm is supported on a chair during the exercise. *Light blue arrows* represent trunk elongation with cranial and caudal forces. *Green half-moons* represent areas of expansion of the concavities. *Red arrows* represent areas of muscle activation, approximating the convexities towards midline, and the direction of the correction. The *dark blue arrow* pointing upwards from the right elbow represents the shoulder traction, which is an isometric tension from the shoulder in a lateral/outward direction with a fixed scapula as a continuation of the transversal expansion in the proximal thoracic region

The Prone exercise corrects the thoracic curve using shoulder traction (ST) and shoulder counter-traction (SCT) and the lumbar curve via activation of the iliopsoas muscle (Fig. 28). The Sail exercise is a very effective stretching exercise, which helps elongate the thoracic concavity (Fig. 29). The Muscle-cylinder engages the quadratus lumborum muscle to correct the lumbar curve against gravity (Fig. 30). Other exercises related to the Schroth method involve postural correction during activities of daily living. These exercises focus on correcting posture while resting, sitting, or standing.

### Activities of daily living

**Fig. 31:**
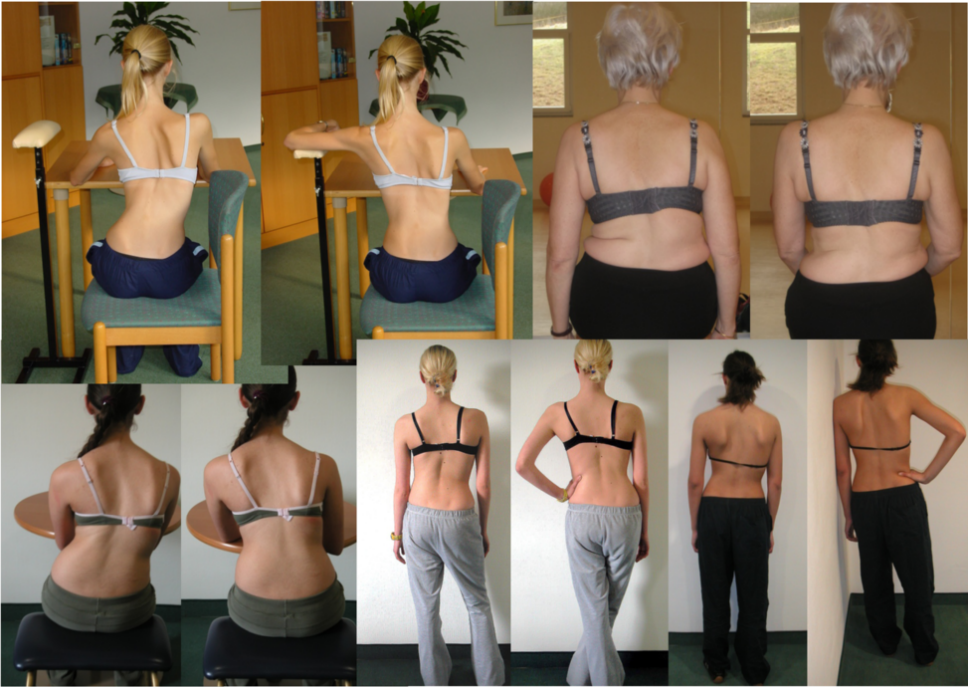
Patients performing Schroth 3D postural corrections in sitting and standing positions. These postural corrections are practiced during activities of daily living in order to change habitual default postures and improve alignment, pain, and curve progression

The Schroth method emphasizes teaching postural corrections throughout the day in order to change habitual default postures and improve alignment, pain and progression (Fig. 31). The main advantage of this program lies in its application to ordinary daily activity for the purpose of changing the asymmetrical loading on the body in order to decrease progression and pain. This also reduces the amount of time needed to practice the highly demanding exercises and allows patients to spend more time in leisure activities and to live a normal life.

### Scientific evidence

Among all PSSE approaches, the Schroth method [14] is among the most studied and widely used specific exercise approaches for scoliosis. Numerous studies have been written by Dr. Hans Weiss, the medical director of the Asklepios Katharina Schroth Rehabilitation Center from 1995 to 2008, and by Dr. Manuel Rigo, director of the Barcelona Scoliosis Physical Therapy School (BSPTS). Their studies [15–29] demonstrate positive outcomes from use of the Schroth method on back muscle strength, breathing function, pain, quality of life and self-image, slowing curve progression, improving Cobb angles and decreasing the prevalence of surgery.

A recent study by Kuru et al., suggests that Schroth exercises performed in a clinic under supervision are superior to home exercise programs only, with results indicating significant improvement in Cobb angle, quality of life and trunk rotation [7]. A study by Schriber et al., confirms in a RCT improved self-image and quality of life in patients that were assigned to a Schroth exercise group as compared to a control group [1]. Another study that followed the Schroth principles and the BSPTS protocol showed an improvement in back asymmetry and spinal imbalance both in the frontal plane and in the transversal plane [30]. The Schroth method has been shown to positively influence the Cobb angle, vital capacity, strength and postural defects in AIS [31]. Furthermore, in reducing the proportion of children with AIS requiring surgery, conservative methods of scoliosis treatment should never be ruled out from scoliosis management as they afford patients a viable alternative to surgical treatment [15].

## Scientific exercise approach to scoliosis (Italy)

### Introduction

The Scientific Exercise Approach to Scoliosis (SEAS) is an individualized exercise program scientifically adapted to all aspects of the conservative treatment of scoliosis based on the most current research, and is continuously evolving with the introduction of new knowledge from the scientific literature. For mild-moderate curves during active growth, SEAS is used alone to reduce the need for bracing. In moderate-severe curves during active growth, SEAS is used in combination with bracing in order to slow down, halt, and possibly reverse curve progression, and in preparation to wean the patient off the brace. In adult scoliosis patients, either with progressive scoliosis curves or fused spines, SEAS helps stabilize the spine and reduce disability.

The SEAS method [32] is based on a scoliosis-specific active self-correction technique performed without any external aids and incorporated into functional exercises. Evaluation tests guide the choice of the exercises most appropriate to the individual patient. Improvement of the stability of the spine in active self-correction is the primary objective of SEAS. SEAS exercises train neuromotor systems to activate a reflex of self-correction of posture during activities of daily living. SEAS can be performed as an outpatient (2–3 times a week for 45 min) or as a home exercise program of 20 min daily in conjunction with expert physiotherapy sessions of 1.5 h every three months for continuous assessment and the adapted modification of the therapeutic program.

### History

**Fig. 32:**
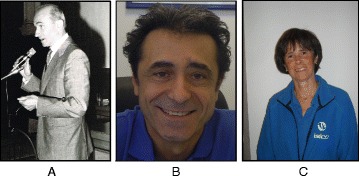
Scientific Exercise Approach to Scoliosis (SEAS) school leaders Antonio Negrini (**a**), Michele Romano (**b**), and Alessandra Negrini (**c**)

The SEAS method [36] originated with the Lyon approach of conservative scoliosis treatment. In the early 1960’s, Antonio Negrini (Fig. 32a) and Nevia Verzini founded a scoliosis center that later became known as the “Centro Scoliosi Negrini” (CSN) in Vigevano, Italy. In 2002, the name was changed to the Instituto Scientifico Italiani Colonna Vertebrale (ISICO), or the Italian Scientific Spinal Institute, which taught the SEAS approach based on scientific principles. Today, Michele Romano and Alessandra Negrini (Fig. 32b-c), both physical therapists and the developers and trainers of the approach, are the leaders of the school, treating and educating around the world.

### The SEAS method

The SEAS method is a scoliosis treatment method that focuses on regaining postural control and improving spinal stability through exercises involving active 3D self-correction of the scoliotic posture. Active 3D self-correction is accomplished first through patient education and increasing the patient’s awareness of their deformity. Once the patient is aware of their deformity and the changes required correcting it, the patient is able to consciously make adjustments to their posture (active self-correction) to find the best possible alignment of the spine within 3D spatial planes. The SEAS method then focuses on spinal stabilization and posture maintenance through a variety of exercises according to the physiotherapeutic literature to help achieve eventual subconscious self-correction of posture through stimulation of neurosensory mechanisms of posture maintenance. Active 3D self-correction can be replicated in a thousand different exercises with “distracting” situations that place demand on neuromuscular connections to increase stability while performing movements, daily actions, and exercises such as sit-to-stand, ascending and descending stairs, balancing on one leg or reaching with the arm above the head, thereby “strengthening” the neuromuscular connections involved in posture correction and neuromotor rehabilitation (active exercises to learn behavior).

Another very important element of the SEAS method is the “team approach” involving the physician, the physical therapist, the orthotist, and the patient’s family. This approach is based on the belief that teamwork produces greater success in treating such patients than the work of a single professional. Teamwork improves patient compliance with exercises, leading to an improved outcome. Family counseling, with strong involvement from all family members throughout the treatment course, is also an important aspect of the SEAS treatment plan.

### Classification system

The first attempt to develop a classification system for idiopathic scoliosis was made in 1950 by Ponseti and Friedman. Ponseti and Friedman developed a classification system for idiopathic scoliosis in 1950 based on the number of curves and the location of the curves. In their classification, idiopathic scoliosis was classified as single curve, double curve, or triple curve. These curve patterns were then described based on the location of the curve apices – cervico-thoracic, thoracic (apex above T12-L1), thoracolumbar (apex at T12-L1), and lumbar (apex below T12-L1), and combined double primary.

Double curve scoliosis has a higher risk of progression than single curve scoliosis, and thoracolumbar and lumbar curve patterns have higher risks of progression than thoracic curve patterns. Although fundamental to classification, curve type and location alone do not accurately describe the complex 3D deformity. Moreover, this strict classification system does not account for the fact that these curves are dynamic, constantly changing in size and location as the patient with scoliosis grows. Later idiopathic scoliosis classification systems have been developed to address these shortcomings. Accurate scoliosis curve descriptions are important in deciding treatment.

### Treatment indications and treatment goals

As with the other scoliosis treatment methods, the indications for scoliosis treatment with the SEAS method are based on the SOSORT guidelines. The primary therapeutic goal of the SEAS method is to increase spinal stability. Other goals include developing postural balance, preservation of the physiological sagittal orientation, halting and even possibly reversing Stokes’ ‘vicious cycle’ of curve progression, and improving vital capacity and quality of life.

The SEAS method can also be used in AIS patients wearing corrective braces. An generalized exercise program helps activate muscles to stabilize the spine and to stimulate ventilation in the lungs. Once the brace is removed, the patient is able to maintain a corrected posture due to increased core muscle strength, improved vital capacity and maximized oxygen intake. Physical exercise, therefore, helps reduce impairments and disabilities due to orthotic wear. Furthermore, because braces induce a “negative body image” in growing children and adolescents, which in turn leads to illness, low self-esteem and psychological problems, exercises help reduce disability induced by wearing the brace and the patient’s feeling of inferiority compared to their friends. More specifically, an exercise program increases the corrective forces exerted by the brace. The idea is that exercises are “dynamic tools” and amplify the “static” forces applied by the orthosis. Exercises also help prevent muscular hypotrophy caused by immobilization of the ribs and spine by the brace by exercising these muscles during bracing.

**Fig. 33:**
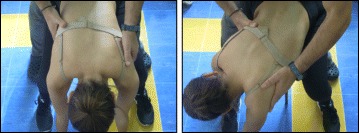
Right thoracic curve mobilization in preparation for bracing is aimed at increasing the range of motion of the spine according to the SEAS method

**Fig. 34:**
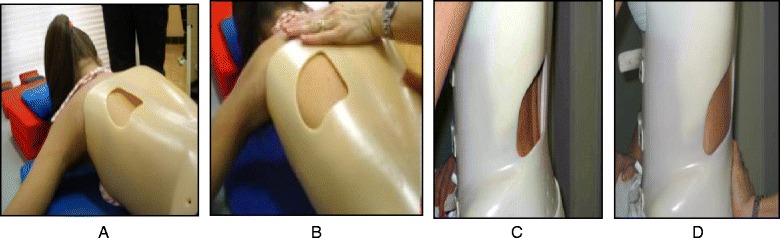
SEAS exercises in brace. The patient is in a relaxed position lying prone (**a**) and then lifts the trunk away from the sternal part of the brace to increase the thoracic kyphosis (**b**). Similarly, the patient is in a relaxed standing position (**c**) and moves the abdomen posteriorly away from the abdominal part of the brace to increase the force on the lumbar pressure pad (**d**)

The SEAS method can be used in preparation for bracing (Fig. 33), during the brace-wearing period (Fig. 34), and during brace weaning. Prior to bracing, SEAS is recommended to increase the range of motion of the spine along all planes so as to allow the brace to exert the maximum possible correction; these mobilization exercises are to be continued during the first phase of brace wearing. During the brace-wearing period, modeling exercises to increase brace pressure on the humps as well as muscular endurance strengthening exercises, requiring lumbar lordosis and thoracic kyphosis preservation, are recommended. Breathing activation exercises are recommended when significant reductions in vital capacity are detected.

### Age specifics

Regardless of the age of the scoliosis patient, the treatment objective is the same: slow down and/or halt curve progression. In children and adolescents, active 3D self-correction is the key to treatment in order to reduce the progressive deformation of the vertebrae while the spine is still growing. Because bone plasticity ends at the end of skeletal growth and bone vertebral deformities are fixed, the primary treatment goal in adults is not to realign the spine and reduce the magnitude of the curve, but rather, to stabilize the spine and prevent further curve progression. Although all adult patients still perform active 3D self-correction, the purpose of these exercises is not to reduce curve magnitudes as in children and adolescents, but rather to stabilize the spine and prevent curve progression.

### 3D principles of correction

Active 3D self-correction with SEAS requires that the patient ask themselves four questions and respond accordingly:

1. “Is my spine supported and not relaxed?”
  While performing SEAS exercises, patients are always told to start from where the spine is in a position of basic support. Once the patient is aware that their spine is supported and not relaxed, the patient then performs the self-correction first with the assistance of a mirror and later without.
2. “Is my body more symmetrical than before?”
  To verify that they have successfully performed the self-correction, the patient must ask if their body is more symmetrical than before. Because the patient initially performs self-correction in front of a mirror, the first test is visual (I *see* that my body is now more symmetrical than before). But over time, as the patient becomes more in-tune with their sensory-motor perceptions, they are able to *feel* that their body is more symmetrical than before and are able to perform exercises without the help of a mirror.
3. “While doing the exercise, am I able to maintain the correction?”
  The answer to this question helps the therapist adjust the level of difficulty of the exercises. If the patient is able to maintain the correction, the therapist may decide to increase the difficulty of the exercise. If the patient is unable to maintain the correction, the therapist will know that the patient should perform an exercise that is less difficult.
4. “Am I able to recognize that my body returns to the original position that it was in before performing the self-correction?”
  The patient performs the exercise for about ten seconds, then slowly relaxes, returning from the self-corrected position to their normal position. By answering “yes” to this question, it means that the patient was able to observe a change in position from the self-corrected position to the usual relaxed position. This question is very important to verify that the exercise was carried out properly. If the patient answers “no” to this question, that means that the self-correction was lost at some point during the execution of the exercise, and the exercise performed has lost its corrective specificity. If the patient is unable to correctly perform the exercise because the patient finds the exercise too difficult to maintain, then the self-corrective exercise should be changed to a less demanding one until the patient is able to answer “yes” to all four questions pertaining to a specific exercise.

### The use of breathing mechanics, muscle activation, and mobilization

**Fig. 35:**
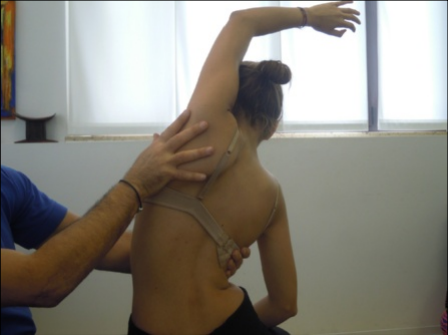
SEAS mobilization and flexibility exercises of the spine to improve joint mobility for better posture correction

Controlled breathing mechanics help with the corrective movements. Muscle activation helps to stabilize the trunk and maintain the correct alignment. Stabilization of the trunk is one of the primary objectives of SEAS. Exercising the muscles helps achieve self-correction during activities of daily living. Mobilization and flexibility exercises of the spine and other parts of the body are also important (Fig. 35).

### Active and passive assistive devices during exercise

**Fig. 36:**
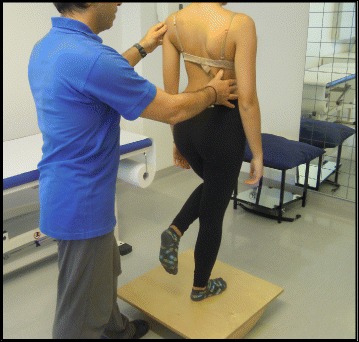
Assistive devices like balance boards are used at the beginning of learning the SEAS method

Assistive equipment such as balance boards (Fig. 36) is used only at the beginning of SEAS to help the patient achieve more effective self-correction; later, it is removed. The mirror is the only tool that helps the patient with active self-correction during SEAS.

### Description of the most relevant exercise mechanics

One of the principle differences between the SEAS method and other methods of scoliosis treatment is that there is no single exercise that is considered better than the others. The goal of SEAS treatment is postural rehabilitation through increasingly difficult exercises that challenge the patient to achieve and maintain active self-correction. Through a sequence of corrective movements specific to a patient’s curve type, the patient is challenged to attain a spinal alignment that is as physiologic as possible. Active self-correction along three spatial planes is the most important component of SEAS. For the choice of the direction of the self-correction, the SEAS method tries to adapt the concept to the patient. This means that in the SEAS approach, there is not a defined sequence of self-correction movements but rather an individual choice of adapted self-correction that is based on the radiographic and postural evaluation, as well as on observed asymmetries.

The goal of treatment is to stimulate a reaction against the deviation. This reaction cannot be properly invoked unless the patient has been able to train themselves properly. It is not useful to set up a self-correction exercise that is theoretically “better” for a specific scoliosis case if the patient is not able to perform it properly and hold it for the required length of time. It is important to settle for a simpler movement that the patient performs correctly and then to focus gradually on an increase in exercise difficulty. Once the patient has successfully learned the correct movements, the active self-correction is performed by the patient independently and then applied to every exercise the patient performs.

**Fig. 37:**
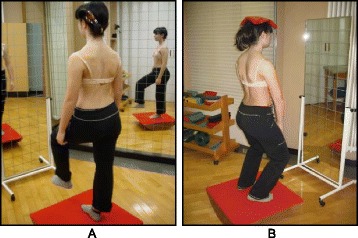
SEAS exercises aimed to improve balance while maintaining active self-correction either by standing on one leg on a balance board (**a**) or by performing a knee-bending exercise on the balance board (**b**)

**Fig. 38:**
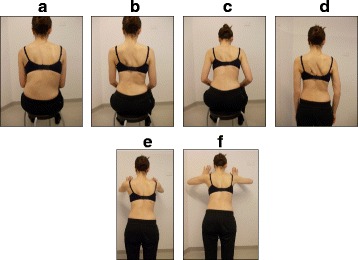
SEAS principles of maintaining self-correction during activities of daily living such as sitting (**a**), sitting leaning forward in preparation for standing and sit-to-stand (**b**, **c**), standing (**d**), and landing on a wall (**e**, **f**)

SEAS also focus on muscular endurance and strengthening in the correct posture, development of balance reactions (Fig. 37), and neuromotor integration. Muscle endurance strengthening aims at developing the paravertebral, abdominal, lower limbs and scapulo-humeral girdle muscles through isometric contractions to increase the muscular support of the spine in order to stabilize the scoliotic spine. The development of balance reactions is aimed at improving axial, static, and dynamic balance of the trunk. This is important in posture rehabilitation because of the impairments in cortical centers of the brain that control balance in scoliosis. Neuromotor integration aims to integrate everyday behaviors with more correct and balanced postures, progressively developing the ability to react with active self-correction to the different requirements of social life, and challenging the patient to maintain the self-correction during activities of daily living (Fig. 38). These exercises associate active self-correction with global movements, e.g., walking with a simple gait and ocular-manual education exercises, even on unstable planes.

### Activities of daily living and sport

**Fig. 39:**
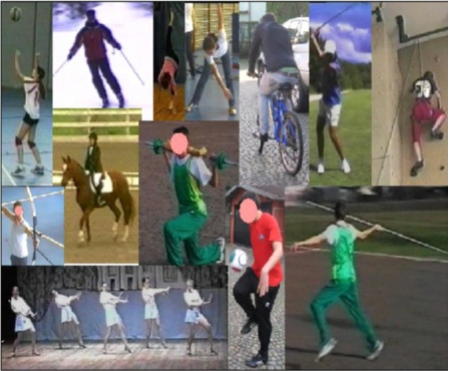
SEAS encourages patients to participate in sports and athletic activities

**Fig. 40:**
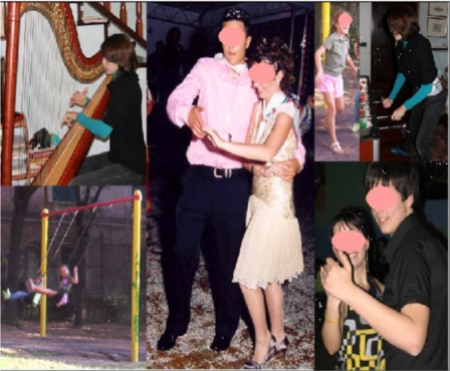
SEAS encourages patients to live a normal life

During complete brace weaning, SEAS teaches ergonomic elements aimed at avoiding spinal damage in adulthood. During the course of the brace treatment, it is of fundamental importance to continually preserve aerobic function and develop a positive body image. For this reason, it is recommended that AIS patients increase participation in athletic activities, professional and/or recreational, even during fulltime bracing (Figs. 39 and 40). The SEAS method holds that the brace should not impose any limitations on a young patient’s personal and social life; it encourages an active lifestyle and promotes a positive body image.

### Scientific evidence

**Fig. 41:**
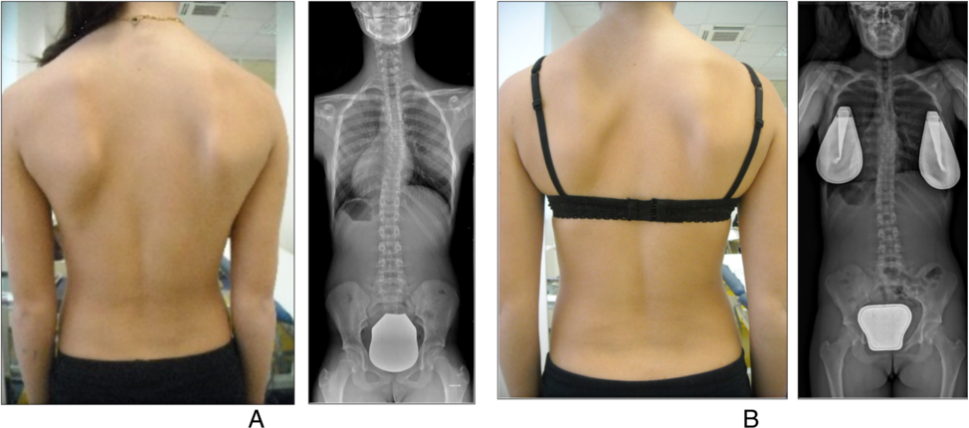
Photograph and radiograph of a patient with scoliosis before SEAS (**a**) and 24 months later after 2 years of SEAS without bracing (**b**)

**Fig. 42:**
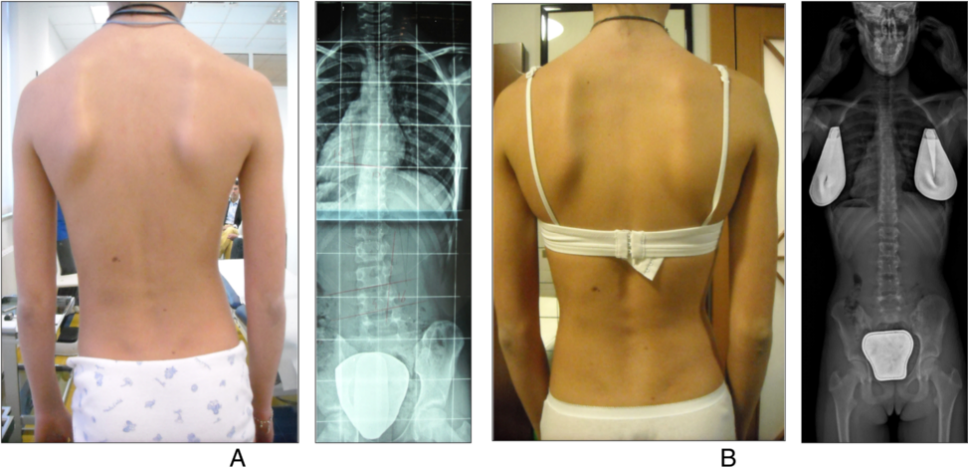
Photograph and radiograph of a patient with scoliosis before SEAS (**a**) and 43 months later after 3.5 years of SEAS without bracing (**b**)

A study in 2008 [33], designed to confirm whether the indication for treatment with specific exercises for AIS has changed in recent years, found that with only a single exception, all studies confirmed the efficacy of exercises (Figs. 41 and 42) in reducing the progression rate (mainly in early puberty) and/or improving the Cobb angle (around the end of growth). One RCT (mentioned in the above 2008 review) showed improvement of curvature in all treated patients after six months; the exercises were also shown to be effective in reducing the need for brace prescription and surgery. Another paper from 2008 set out to compare the effect of SEAS exercises with “usual care” rehabilitation programs and confirmed the effectiveness of exercises in patients with scoliosis who are at a high risk of progression and that compared with non-adapted exercises, a specific and personalized treatment (SEAS) appeared to be more effective [33]. Other papers supported the SEAS approach to scoliosis exercise treatment concluding that SEAS exercises can reduce bracing and in the case of patients wearing the brace, they assure the maintenance of the correction achieved [34].

Moreover, SEAS has a strong modern neurophysiological basis, to reduce requirements for patients and possibly the costs for families linked to the frequency and intensity of treatment and evaluations. Therefore, SEAS allows for treatment of a large number of patients coming from far away [32]. Furthermore, exercises can help reduce the correction loss in brace weaning for AIS [32].

### SEAS braces

#### SIBILLA BRACE (Fig. 43)

**Fig. 43:**
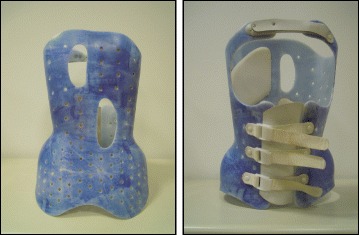
The Sibilla brace. Designed for mild progressive AIS, the Sibilla brace is prescribed for scoliosis curves up to 30° Cobb angle

In mild progressive AIS (up to 30° Cobb) that cannot be controlled through SEAS exercises, the first aim is to avoid progression while allowing the maximum possible freedom in activities of daily living and reducing the discomfort caused by the brace. In such cases, the chosen brace will be less rigid (Sibilla) and will have to be worn for 18 to 20 h each day until the end of the progressive period (up to Risser stage 3), at which point the patient will be weaned off the brace.

#### SFORZESCO BRACE (Fig. 44)

**Fig. 44:**
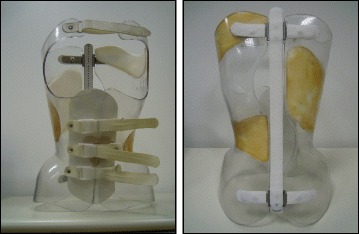
The Sforzesco brace. Designed for severe scoliosis in adolescents, the Sforzesco brace is prescribed for scoliosis curves up to 45°–50° Cobb angle or more if surgery is not an option

In severe adolescent scoliosis (up to 45°–50° Cobb, and over if the patient does not want to be operated on or if surgery is not a viable option), the aim is, at minimum, to avoid progression (and surgery) [35], and possibly even to reduce the magnitude of curvature; however, this does not guarantee stability in adulthood. In these cases, a brace is worn for the entire day for at least one year, and the most rigid brace is chosen (Sforzesco). Afterwards, brace wearing is gradually reduced by one or two hours every six months, while maintaining the results, even if the brace must be worn up to 18 h a day, until Risser stage 3.

## Barcelona scoliosis physical therapy school (Spain)

### Introduction

The Barcelona Scoliosis Physical Therapy School (BSPTS) is based on the principles developed by Katharina Schroth [14], and is used primarily to treat AIS, certain forms of congenital scoliosis, and sagittal deformities such as Scheuermann’s disorder. The indications for PSSE are oriented to the particular patient. Treatment is based on an integral scoliosis care model, which includes specific education, observation or surveillance, psychological support and intervention, bracing in accordance with Rigo-Chêneau principles, and surgery. Diagnosis and patient evaluation are essential in this model aimed at patient-centered decision-making according to clinical experience, external evidence and the patient’s preference. Thus, specific exercises are not considered as an alternative to bracing or surgery but as a therapeutic intervention, which can be used alone or in combination with bracing or surgery according to individual indication.

### History

**Fig. 45:**
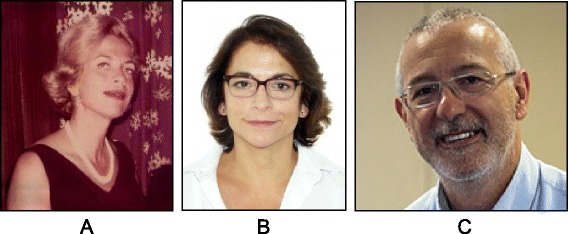
(**a**, **b**, **c**): The BSPTS founders Elena Salvá (**a**), Dr. Gloria Quera-Salvá (**b**), and Dr. Manuel Rigo (**c**)

The predecessor to BSPTS was founded in 1968 in Barcelona, Spain, by Spanish physiotherapist Elena Salvá (1926–2007) (Fig. 45). The school adopted the Schroth principles and the original intensive inpatient rehabilitation exercise program of the Katharina Schroth Clinic in Bad Sobernheim, Germany. Elena Salvá met Katharina Schroth and her daughter, Christa Lehnert-Schroth, creators of the Schroth method, in Germany during the 1960’s. Salvá became close friends with Schroth and Lehnert-Schroth, who taught Salvá about the Schroth method for the conservative treatment of scoliosis. Salvá returned to Spain with a new perspective on scoliosis treatment and founded the Elena Salvá Institute for the conservative treatment of spinal deformities in Barcelona. Salvá was dedicated to the treatment and rehabilitation of patients with scoliosis and other spinal deformities, such as kyphosis. She used the Schroth method for more than forty years before her passing in 2007.

In 1989, Elena Salvá’s daughter, Gloria Quera-Salvá, and Manuel Rigo (Fig. 45) began educating and certifying Spanish physiotherapists under the Schroth method. By 2001, physical therapists from the United States, Israel and many other countries were traveling to Barcelona to receive certification under the Schroth method. With the contribution of several experienced Spanish physiotherapists, but in keeping with the same basic Schroth principles as used in Germany, BSPTS created its own modified method of scoliosis physiotherapy. By 2009, BSPTS began certifying physiotherapists under the new BSPTS method. The first International Body of Instructors for the school was formed in 2011 and now offers scoliosis rehabilitation education courses under the BSPTS method to physical therapists around the world.

### About the method

BSPTS is a physiotherapeutic method that can be defined as a therapy plan of cognitive, sensory-motor and kinesthetic training to teach the patient to improve their scoliosis 3D posture and shape based on the assumption that scoliosis posture promotes curve progression, according to the ‘vicious cycle’ model [36]. It adheres to the original principals of Katharina Schroth, providing 3D treatment based on breathing and muscle activation.

The method recommends that physical therapists work as part of a multidisciplinary team in accordance with the SOSORT guidelines and the philosophy of the Scoliosis Research Society (SRS). This philosophy considers the human element involved in the treatment of scoliosis, and it stresses the importance of not introducing false fears to patients diagnosed with mild, non-progressive or stable scoliosis in order to make them long-term clients of the physiotherapy clinic.

### Classification system

**Fig. 46:**
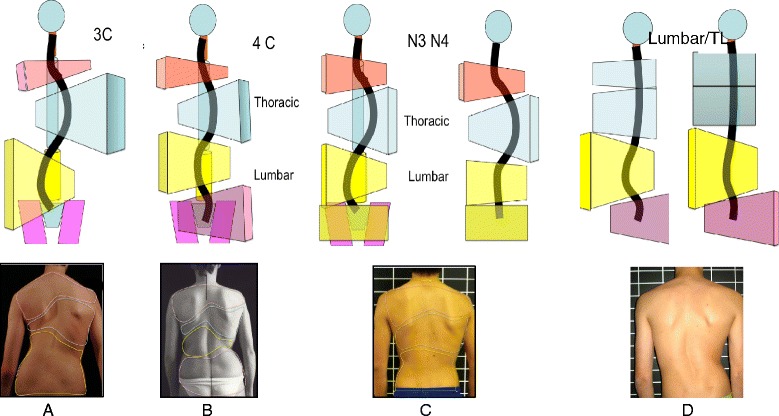
(**a**, **b**, **c**, **d**): The BSPTS system of scoliosis curve classification illustrated with photographs and body block diagrams. The four scoliosis curve types in this classification system are 3C (**a**), 4C (**b**), N3N4 (**c**), and single lumbar or thoracolumbar (**d**). The 3C curve is a major thoracic scoliosis curve with a compensatory lumbar and pelvic shift (**a**). The 4C curve is a major lumbar scoliosis curve with a thoracic and lumbar shift (**b**). The N3N4 curve is a major thoracic scoliosis with or without a lumbar curve but with the pelvis in a neutral position (**c**). The single lumbar or thoracolumbar curve is a single curve scoliosis with an uncoupled pelvic shift and no thoracic curvature (**d**)

Every type and sub-type of scoliosis is classified in accordance with a schema of blocks (Fig. 46) or trunk regions, which is based on the original Schroth classifications first developed by Katharina Schroth and later modified, in 2010, by Manuel Rigo [16]. The blocks illustrate the patient’s spinal curve pattern by showing the shifts and rotations of the scoliotic deformity in three dimensions. By allowing both therapist and patient to visualize the deformity, the blocks assist in educating the patient and creating an appropriate plan to treat the patient.

The classification system includes three basic groups labeled 1, 2, and 1–2, where Group 1 represents sagittal deformities and Group 2 and Group 1–2 represent scoliosis, and here is their description:

1. **Group 1** describes sagittal deformities such as hyperkyphosis (mainly due to Scheuermann’s Kyphosis), inverted back (hypokyphosis), and flat back.
2. **Group 2** defines a structural scoliosis in the main thoracic region, with no lumbar curve or combined with a minor functional or minor structural or major structural lumbar or thoracolumbar curve. Group 2 can be subdivided into three different patterns: 3 Curves, 4 Curves and non 3–non 4.
  - Three-curve scoliosis pattern (3C) means a major thoracic curvature with a major structural lumbar curvature that is combined with the pelvis. The lumbar spine and the pelvis function as one unit in the schema of body blocks, and will shift and rotate to the opposite side of the thoracic curvature.
  - Four-curve scoliosis pattern (4C) is a major lumbar curvature with a compensatory thoracic curvature and a pelvis that shifts and rotates to the opposite side of the lumbar curvature.
  - Non 3-non 4 (N3N4) is defined by a major thoracic curvature with or without a lumbar curvature with a pelvis that is not shifted and not rotated, i.e. one that is balanced in the center.
3. Group 1-2 defines a lumbar or thoracolumbar curve with a rectilinear thoracic spine.

**Fig. 47:**
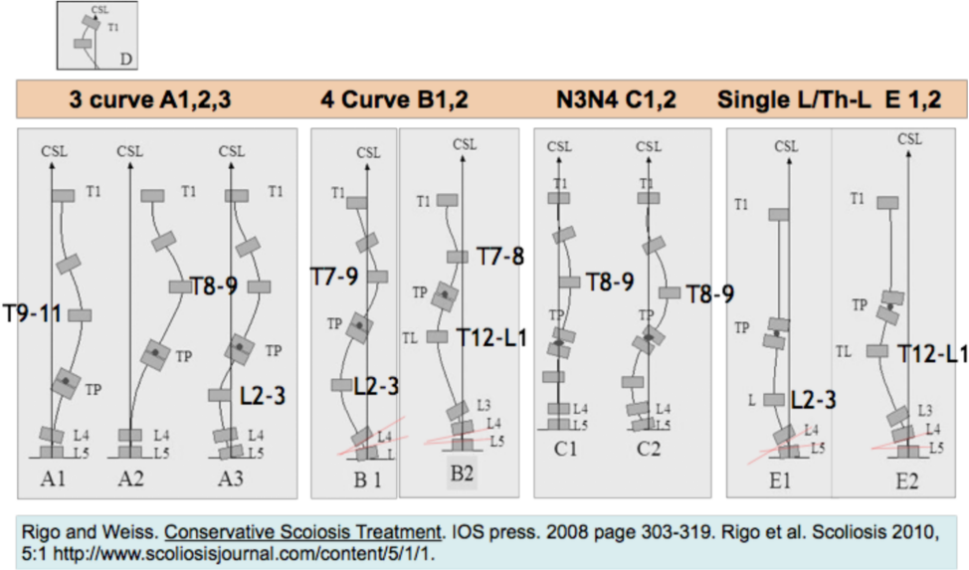
Rigo classification for BSPTS bracing and physical therapy

Rigo’s Radiological Classification System [16] uses objective radiological criteria to confirm the functional curve type (Fig. 47). This current classification system was developed specifically by Dr. Rigo in 2010 to correlate with brace design and physiotherapy [16]. Patients must be classified as 3C, 4C, N3N4 (Group 2) or single lumbar/TL (Group 1–2), as described above, based on clinical observation. Later the radiological criteria are used to confirm the initial clinical diagnosis. From a clinical perspective, Group 2 correlates to A, B and C types, respectively, in the radiological classification. A, B and C types can be at the same time subdivided as A1, A2, A3, B1, B2, C1 and C2. Group 1–2 correlates to E type (E1 and E2) in the radiological classification. The presence of a structural curve in the proximal thoracic region is defined as ‘D modifier’.

### Treatment indications

Indications for treatment are outlined in the SOSORT guidelines [9] and focus primarily on the conservative treatment available to prevent curve progression. The BSPTS method is designed specifically for physiotherapists. The physiotherapist requires extensive training and many clinical years of experience in order to perfect the BSPTS method. There are some elements of the BSPTS method that may benefit patients with other spinal deformities, but the BSPTS approach has been used primarily for idiopathic scoliosis (late JIS and AIS). Other types of scoliosis may be treated with modified principles. Sagittal plane deformities such as hyper-kyphosis (Scheuermann’s kyphosis) and lordosis (inverted back) can also be treated with Schroth exercises. A modified Schroth program is used to treat painful degenerative adult scoliosis. BSPTS principles, but not the full active plan of exercises used classically for adolescents or adults, can be used in early onset scoliosis.

### Goals

**Fig. 48:**
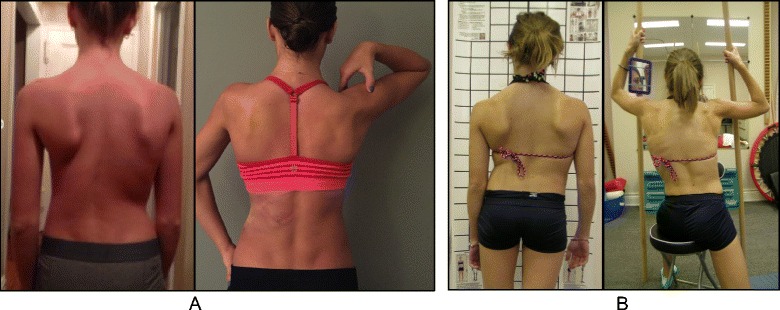
(**a**, **b**): Active 3D self-correction exercises. During active 3D self-correction, patients expand the collapsed areas and open the concavities by performing rotational angular breathing (RAB) and specific arm positions (**a**). During the Schroth-derotation sitting exercise (**b**), the patient sits on a chair, with a pole in either hand planted on the ground, while performs corrections 1–5, while stabilizing her curve specific corrections. (48b provided with the permission of Andrea Lebel, RPT, MCPA, Ottawa, Canada)

The goals of the BSTPS method are to 1) correct the ‘scoliotic posture’ (Fig. 48) and improve aesthetics, 2) stabilize the spine and arrest the curve progression, 3) educate patients and families about the condition and treatment options, 4) improve breathing function, 5) increase activity, including activities of daily living and functional mobility, 6) improve overall self-image and self-esteem, and 7) decrease pain. The higher the risk of curve progression, the more intense the conservative treatment plan should be in order to meet the goals of therapy. However, this objective should not delay the recommendation for bracing or surgery when indicated. BSPTS is not an alternative to or substitution for bracing or surgery and has its own indications.

### 3D Principles of correction

**Fig. 49:**
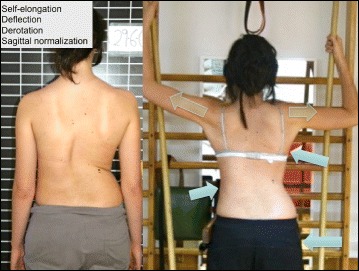
Patient with a major left lumbar-thoracolumbar scoliosis curve with a right pelvic shift performs a two-pole standing exercise applying the BSPTS principles of correction 1–5. *Light brown arrows* represent bilateral shoulder traction, which is required for stabilization during active self-correction. *Light blue arrows* represent bilateral shoulder counter-traction, which is required for midline spinal alignment. The *light blue arrow* pointing to the patient’s pelvis represents pelvic correction from the right to the midline, which is required exercise when the patient has a major lumbar or thoracolumbar scoliosis curve

BSPTS principles of correction are based on the original principles described by Katharina Schroth [14]. The treatment is individualized depending on the curve type (described in About the method above) and is done only after the individual has achieved their best global postural alignment by organizing the lower extremities, the pelvis and the trunk in the best possible posture. The principles of correction follow the global postural alignment and are applied with high intensity forces created inside the body (‘from inside’) involving isometric tensions, expansions and specific breathing. The end result (Fig. 49) is a corrected posture where the collapsed areas of the trunk (the concavities) are open and expanded and the prominences (the convexities) are contained.

The following is a detailed description of the principles:

1. 3D postural correction is made through movements of translation, rotation and mixed (sagittal expansions). The correction follows a schema of blocks, which is based on the classification of functional types first developed by Schroth and later modified by Rigo. The blocks are deformed, translated and rotated in accordance with the spinal curve pattern, and the 3D postural correction is referred not only to a combined but to a real synchronized correction of the position (translation and rotation) and shape (deformity) of all the blocks. Thus, the applied principles of correction can be described as deflection, derotation and sagittal normalization.
2. The expansion/contraction technique (Fig. 50) is used to achieve the ‘best possible correction.’ It facilitates the so-called ‘corrective breathing.’ The best possible correction is only possible, at the beginning of the therapy, with the help of some external aids, including passive and passive-active manual aids offered by the physical therapist. Expansion/contraction technique is about expanding any part of the trunk in any direction ‘from inside,’ by using only the muscle force (independent of breathing movements). The expansion can be side to side or one side against a fixed point. Only the collapsed areas of the trunk will be expanded, while the prominences will be contracted. This technique facilitates the later introduction of ‘rotatory corrective breathing.’ The overall goal is not only to expand and breathe into the collapses or concavities, but to do it in a corrective direction according to well-described biomechanical rules.

**Fig. 50:**
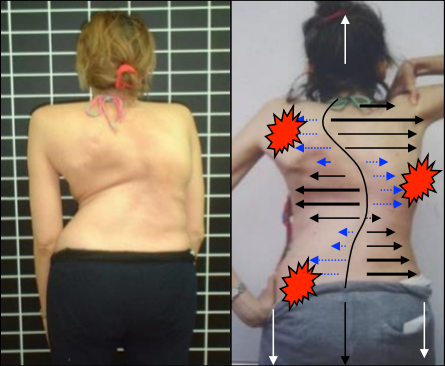
Patient using postural correction and the corrective expansion/contention technique to achieve the best possible correction. The *blue* and *black arrows* represent trunk expansion during the first principle of correction. Later, the *blue arrows* are converted into forces represented by the *red shapes*, which work around the prominences to move the prominences forward and inward
3. Stabilization by muscle tension. Once the best possible correction has been achieved in any specific starting position (the starting positions can vary in accordance with the above described functional types), the subject will be asked to produce muscle tension in order to maintain the correction. Thus, muscle tension can be defined as isometric tension. The maintenance of the correction during this part of the therapy, by creating muscle tension, will produce an isometric eccentric contraction of the previously shortened muscles and concentric contraction of the previously over-elongated muscles. Before creating the tension, the muscle balance has been improved and the new achieved balance is not lost when creating this final muscle tension.
4. Integration. After the exercise, the subject is asked to relax while still maintaining the 3D postural self-correction. Eventually coming again into the best possible correction or going back into the bad posture, the patient notes (proprioception) and sees (indirect by mirror or direct by camera-screen) the differences between scoliotic posture, 3D postural self-correction and ‘best possible correction.’ Repetition of the exercises and the integrative strategies allow the subject to bring the correction into activities of daily living.

### The use of breathing mechanics, muscle activation, and mobilization

**Fig. 51:**
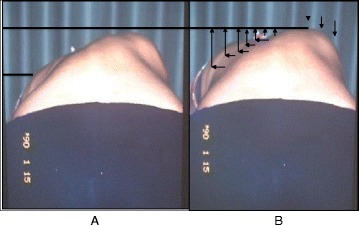
(**a**, **b**): Before (**a**) and during (**b**) rotation angular breathing (RAB). The *arrows* represent directional breathing used to fill the collapsed lungs with air and reshape the thorax (**b**)

**Fig. 52:**
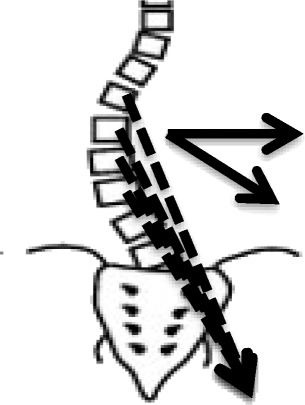
Diagrammatic depiction of the activation of the iliopsoas muscle in a lumbar scoliosis curve. The arrows show the direction of activation from the origin to the insertion points of the iliopsoas, promoting curve de-flexion and derotation towards the right

**Fig. 53:**
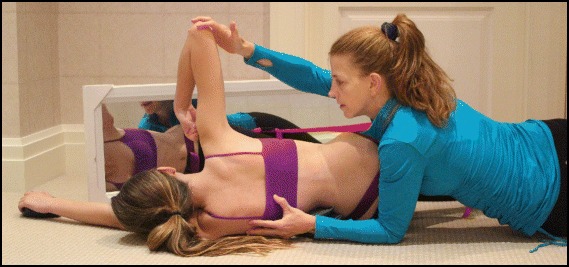
Physical therapist, Dr. Hagit Berdishevsky, assisting a patient in mobilizing the collapsed ribs on the left concave side and expanding the ribcage in an outwards and backwards direction

The success of the method is based on strengthening exercises tailored to each individual scoliosis patient and their specific curve pattern. Unique rotational angular breathing (RAB) exercises (Fig. 51) originally developed by Katharina Schroth [14] help in vertebral and rib cage derotation and in increasing vital capacity. This unique breathing technique helps expand the ribs from inside the rib cage by pushing the ribs “sideways and backwards” and helps return the vertebrae closer to their normal, untwisted position. Muscle activation of core muscle groups, like the iliopsoas (Fig. 52), thoracic and lumbar fascicles of the erector spinae and quadratus lumborum, help stabilize and maintain the expanded ribs and derotated vertebral bodies. Encouraging mobilization and flexibility (Fig. 53) helps to release tension and assists in postural correction. Wall bars, pads, poles, belts, straps, mirrors, elastic-bands, dowels, balls, yoga blocks, stools, and foam rollers are equipment commonly used to assist Schroth exercises.

The training period can vary and an individual can be trained on a one-to-one basis or in a group setting. The therapy is always individualized and even in the group setting, every patient is treated individually by the physical therapist. Only very experienced therapists can manage individual treatment in a group session. There are two reasons to hold group sessions: first, the cost of the therapy can be reduced, and second, and more important, the environment created is one of group therapy and support. The number of hours necessary to train the subject to do the exercises with efficacy and safety varies, and depends also on the modality, private or group. The groups are limited but this limitation depends also on the therapist’s experience and capability. Sixty hours in-group and 20 h in private is typically enough to achieve a high level technique, but after a few hours of treatment (e.g. nine hours in group) the patients can reproduce the correction in several starting positions and can start practicing at home. The patient can always improve the level of performance with the help of the therapist, but perfection is not required in order to obtain positive results.

### Description of the BSPTS exercises

One of the important aspects of exercising in different positions is that the individual trunk areas are being challenged to work against or with gravity in different positions. The decision of which exercise position to utilize depends on the needs and goals of the patient, with a gravity-eliminated position to assist the patient in activation of intended trunk musculature and an anti-gravity position to increase endurance and muscle activation.

**Fig. 54:**
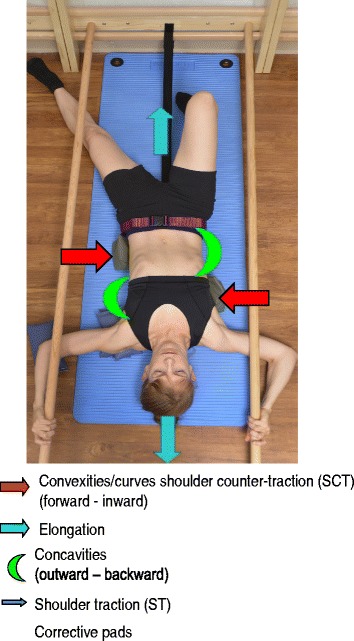
Schroth supine exercise for patients with a major lumbar curve. Turquoise arrows represent the cranial elongation and caudal traction force. The *Green half-moon* represents the area of expansion of the concavity. *Light blue arrows* on the patient’s arms represent bilateral shoulder traction, which is an isometric tension from the shoulder in a lateral/outward direction with a fixed scapula as a continuation of the transversal expansion in the proximal thoracic region. The arm position and muscle activation during bilateral shoulder traction can assist in active self-elongation and in preventing postural collapse. *Red arrows* represent counter traction forces – contraction around the convexities/the curves in a forward and inward direction towards neutral spine

**Fig. 55:**
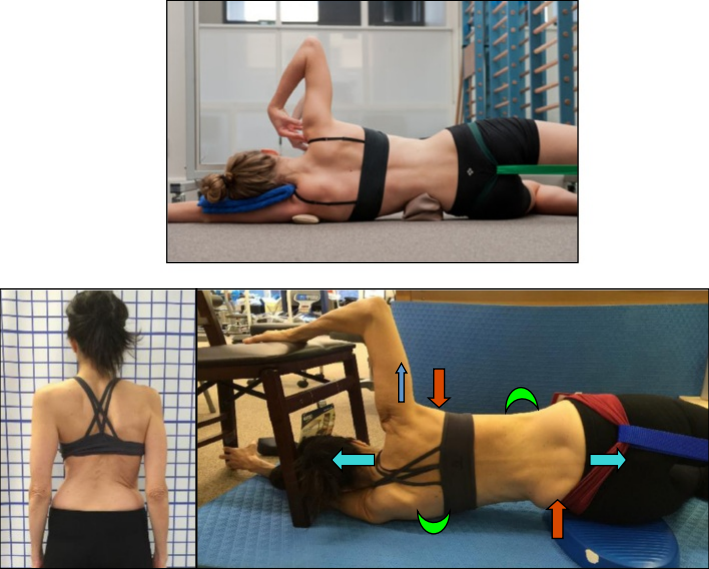
The Schroth “Side-lying” exercise for major lumbar curves (*top*) and major thoracic curves (*bottom*). During this exercise, the patient lies on the lumbar convex side. *Light blue arrows* represent trunk elongation with cranial and caudal forces. *Green half-moons* represent areas of expansion of the concavities. *Red arrows* represent areas of muscle activation, approximating the convexities towards midline, and the direction of the correction to correct the convexities. The *dark blue arrow* pointing upwards from the right elbow represents the shoulder traction

**Fig. 56:**
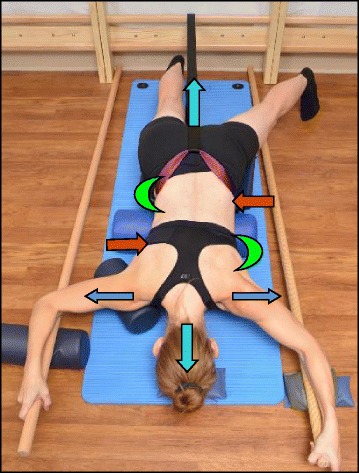
The Schroth prone exercise. The leg of the lumbar convex side (*left leg*) is abducted and the pelvis is supported and elevated by a footstool. The lower abdomen is supported by a roll, as is the right shoulder, to facilitate trunk stabilization during the exercise. *Blue arrows* on the shoulders represent bilateral shoulder traction. Turquoise arrows represent trunk elongation with cranial elongation and caudal traction forces. *Red arrows *represent areas of muscle activation around the convexities towards the midline. *Green half-moons* represent areas of expansion of the concavities

**Fig. 57:**
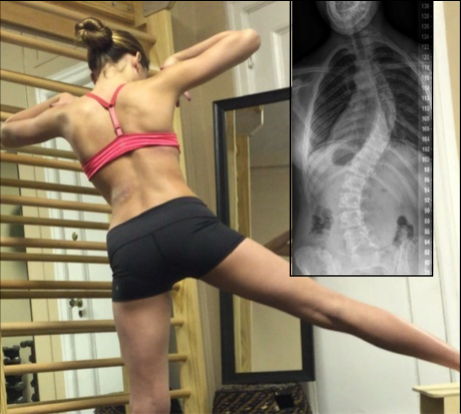
The “Muscle-Cylinder” exercise for major lumbar curves in patients with moderate-severe scoliosis (as seen in the radiograph) helps patients achieve spinal alignment and a corrected posture

Four of the most commonly used exercises in the BSPTS method are Supine exercises (Fig. 54), Side-lying exercises (Fig. 55), Prone on stool (Fig. 56) and the Muscle Cylinder (Fig. 57). The first three exercises can be employed in all of the functional types of scoliosis curve patterns. The ‘muscle cylinder’ exercise is for highly trained patients and is used mainly in the major lumbar (4C) pattern (although there is an old classical version for the major thoracic (3C) curve pattern).

Exercises in the supine position eliminate the force of gravity on the spine so the patient can focus more on small corrections in posture with increased precision. Exercises in the side-lying position are best for corrections in the frontal plane. Additionally, the side-lying position is useful to address a lumbar concavity by placing it upwards and facilitating it against gravity. Exercises in the prone position allow the collapsed areas in the back to work more intensively as they are working against gravity while the prominences have the advantage of facing down and are in a gravity-eliminated position.

The muscle cylinder exercise (Fig. 57) is an advanced level exercise involving very high-level muscle activation against gravity. The entire program of exercises includes many additional exercises, but their description falls outside the scope of this paper. Other exercises related to the BSPTS method involve postural correction during activities of daily living. These exercises focus on correcting the scoliosis posture while sleeping, resting, sitting, or standing, carrying a bag, bending, reaching, and exercising in brace. During these activities, the spine is in a neutral position but the patient focuses on consciously maintaining the correct posture.

### Activities of daily living

**Fig. 58:**
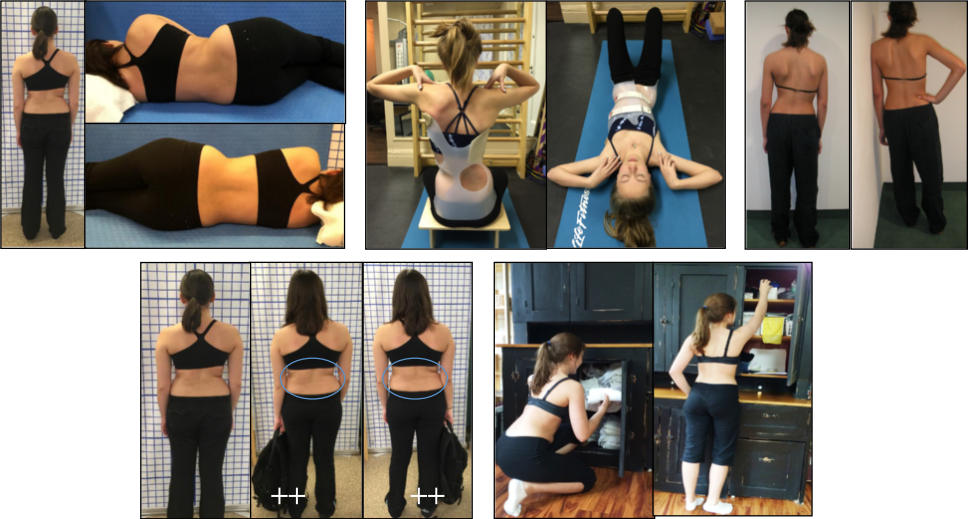
Patients with scoliosis demonstrate how they perform activities of daily living (ADLs) with a proper posture such sleeping, standing, carrying a bag, bending, lifting, and reaching, as well as sleeping and sitting in a brace

**Fig. 59:**
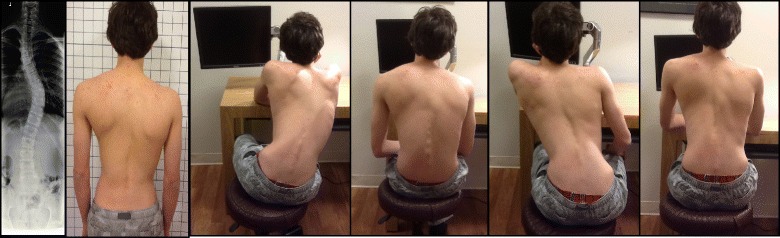
Education and multi-step training is required to achieve the correct sitting posture

The BSPTS believes that it is an important part of its approach to teach patients maintain good posture in all parts of their life. This includes learning how to sit, stand, sleep and move with better alignment and in a specific way based on their unique curve patterns (Figs. 58 and 59). Regarding participation in sports, BSPTS focuses on the entire child/patient and not only on the treatment of scoliosis. BSPTS encourages patients to continue living their lives and to pursue normal psychosocial growth and maturation. This may include a passion for sports, which should be allowed and even facilitated.

### Scientific evidence

BSPTS is based on the principles developed by Katharina Schroth. The scientific evidence may be found in the section describing the evidence for the Schroth method (Scientific evidence).

### BSPTS bracing

**Fig. 60:**
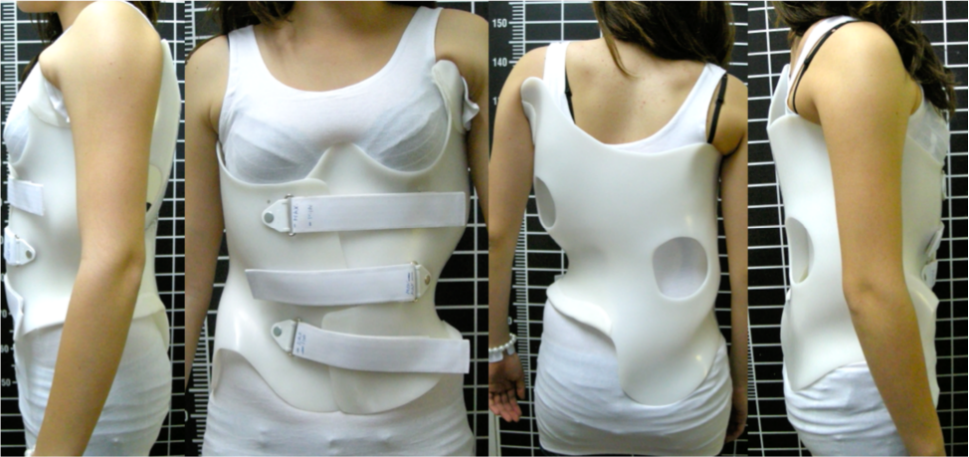
Different views of the Rigo-Chêneau brace. From left to right: later view of the thoracic concave side, frontal, posterior, and lateral view of the thoracic convex side

BSPTS principles are fully compatible with brace concepts such as the Rigo-Chêneau [37] brace type. The Rigo-Chêneau brace (Fig. 60) is a rigid asymmetric custom brace. The original technique was inspired by the plaster cast correction principles. The main objective is to bring the patient into the best possible 3D correction similar to that utilized in a plaster cast, but in this case without any passive traction. A positive plaster mold from the patient is modified in order to define very selective contact areas and correspondent expansion areas. The pads for contact are designed to act at a proper level and with a proper shape and orientation to move the trunk into the best spinal correction. The corrective principle is a combination of three-point systems, regional derotation and sagittal balance, and physiological profile. The lack of compression/”sandwich” effect converts the passive rigid brace into a 4D dynamic brace. Breathing movements, and growth and development, are facilitated in the corrective direction by the brace, so time is essential as a corrective principle as well.

**Fig. 61:**
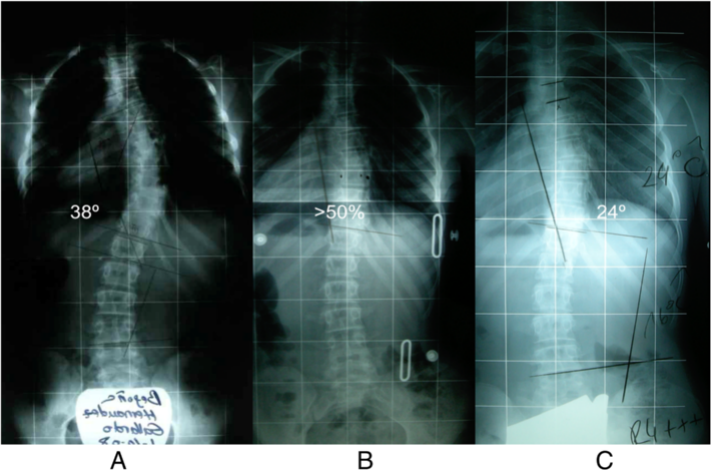
(**a**, **b**, **c**): Series of radiographs of a patient with progressive scoliosis treated with the Rigo-Chêneau brace. **a** The initial radiograph shows a thoracic scoliosis curve of 38° Cobb. **b** In-brace radiograph shows a greater than 50 % scoliosis curve correction. **c** Out-of-brace radiograph at Risser 4 (end of growth) shows that the thoracic curve has been decreased significantly to 24° Cobb angle, a curve reduction of >35 % compared to the initial radiograph

This type of brace can potentially prevent the increase in lordosis commonly observed in full-contact braces working through static pressures rather than corrective movements. The principles of correction applied in the construction of this brace are based on the corrective movements from the BSPTS principles. In Fig. 61 a correction of scoliosis is observed on x-ray.

## The Dobomed method (Poland)

### Introduction

Dobosiewicz’s method for the treatment of scoliosis (Dobomed) is a conservative management approach for idiopathic scoliosis that addresses both trunk deformity and respiratory function impairment. The Dobomed approach has incorporated both Klapp’s position for kyphotization of the thoracic spine [38] and Lehnert-Schroth’s approach for active asymmetrical breathing [14].

### History

**Fig. 62:**
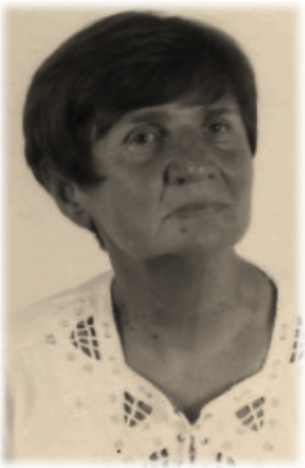
Dr. Krystyna Dobosiewicz (1931–2007), founder of the DoboMed treatment method of scoliosis

The Dobomed method was developed in 1979 by Polish physiotherapist and physician Professor Krystyna Dobosiewicz (1931–2007) (Fig. 62), and has been used routinely in Poland for the conservative treatment of scoliosis since 1982. Prof. Dobosiewicz was very familiar with Klapp’s and Lehnert-Schroth’s methods. It was from these beginnings that Prof. Dobosiewicz started to create her own approach to the treatment of scoliosis. Initially, the Dobomed method was tested on an outpatient group, continuously being improved and modified by Prof. Dobosiewicz before being adopted by the Department of Rehabilitation at the Medical University of Katowice, Poland, in 2000, as an intensive inpatient rehabilitation approach for patients with scoliosis. Since the beginning, the Dobomed approach has been used either as a sole physiotherapy method or in combination with Chêneau bracing [37].

### Definition of treatment

**Fig. 63:**
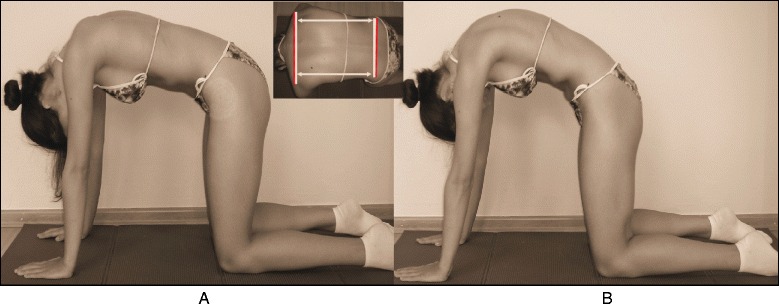
(**a**, **b**): Patient performing typical DoboMed method exercises before the application of thoracic kyphosis (**a**) and with thoracic kyphosis (**b**). Thoracic kyphosis is obtained by fixation of the pelvis and shoulder girdle using the upper and lower limbs

The Dobomed approach is a biodynamic method of 3D auto-correction based on the pathomechanics of idiopathic scoliosis. The basic technique of the Dobomed approach is active 3D correction involving mobilization of the primary curve towards curve correction, with special emphasis on “kyphotization” of the thoracic spine and/or “lordotization” of the lumbar spine [39]. This mobilization of the primary curve is performed in closed kinetic chains and developed upon a symmetrically positioned pelvis and shoulder girdle. The pelvis and shoulders are positioned first and kept stable for the duration of the exercise and during the inspiration and expiration phases of active asymmetrical breathing (Fig. 63). Frontal plane correction occurs automatically as the sagittal and axial planes are corrected. Lateral flexion of the spine is not required for thoracic curves. This symmetrical positioning of the pelvis and the shoulder girdle is something that is unique to the Dobomed method.

### Treatment indications, goals, and age specifics

Treatment indications for the Dobomed method follow the 2011 SOSORT guidelines [9]. Small, moderate, and large scoliosis curves can all be treated with the Dobomed method, either with or without Chêneau bracing.

The goals of the Dobomed method are to stabilize and correct the spinal deformity, and to prevent progression and/or decrease the curvature of the scoliosis. Another aim of this method is to improve the overall functional status of the patient, particularly the respiratory function.

Cooperation is the basic requirement in using the Dobomed method. Therefore, Dobomed is not recommended for small children who are unable to understand and perform the exercises. For older patients, the focus is on stabilization exercises rather than active 3D correction.

### Classification system

The Dobomed method uses its own classification system for scoliosis and scoliosis treatment. Each scoliosis patient has his/her own unique curve pattern and is evaluated separately. A patient’s personalized exercise program depends on the number of primary and secondary curves and the location of these curves.

### 3D Principles of correction

The following are the principle distinctive features of the Dobomed method:

1. Symmetrical positions for exercising.
2. Asymmetrical active movements to accomplish 3D scoliosis correction.
3. Thoracic spine mobilization to increase thoracic flexion.
4. Transverse plane derotation, with specific treatment emphasis focused on the area of the curve apex.
5. Concave rib mobilization to expand and derotate the ribs.
6. External facilitation.
7. Directed movements of the thorax and spine to improve respiratory function.
8. 3D displacement of vertebrae to obtain 3D scoliosis correction.

### The use of breathing mechanics, muscle activation, and mobilization

**Fig. 64:**
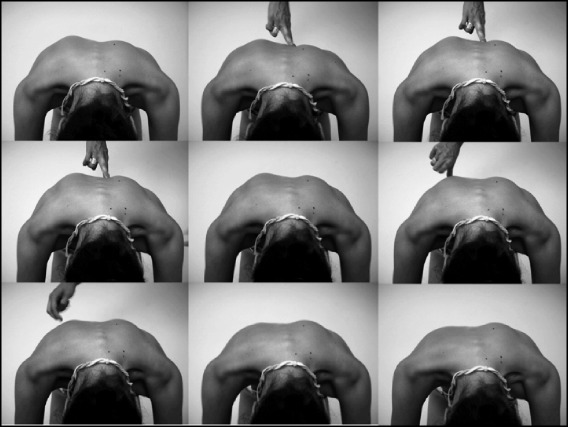
‘Phased-lock’ respiration exercise showing in nine sequential photographs the complete filling of the collapsed lung on the left thoracic concave side. Local subtle pressure applied with a finger facilitates lung expansion

**Fig. 65:**
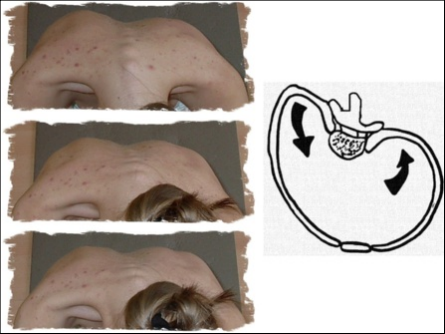
A diagrammatic depiction (*right*) of rotation angular breathing (RAB) exercises with the arrows showing the corrective direction of the ribcage is demonstrated by the patient (*left*) as she expands the lung on the concave side with the goal of reshaping the thorax

The Dobomed method uses the ‘phased-lock’ respiration technique to assist in spinal correction and stabilization. During ‘phased-lock’ respiration [40] (Figs. 64 and 65), a strong local pressure is applied on the concave side during inspiration while a subtle facilitation is applied on the convex side during expiration. During expiration, isometric contraction of the trunk muscles helps to stabilize the correction or hypercorrection. Additionally, activation of the internal intercostal muscles on the convex side during both inspiratory and expiratory phases of breathing allows for asymmetric breathing which results in the ribs coming closer together on the convex side, and mobilization and derotation of the spine.

### Treatment tools: active and passive

The Dobomed method employs the use of mirrors, photographs, and video to assist with scoliosis treatment exercises.

### Description of the most relevant exercise mechanics

The Dobomed method consists of three parts:

1. *Forward bending phase:* Active 3D self-correction of the spine and ribcage during forward bending is an original component and the main corrective technique of the Dobomed method. Forward bending exercises are designed in closed kinetic chains in order to enhance their effectiveness. Forward bending is done with strict fixation of the pelvis and the shoulder girdle with the upper and lower limbs. This bending position is thought to facilitate active self-correction between two symmetrical and stable zones, and helps to consolidate the correct postural habit beyond the therapeutic session.
2. *Preparatory phase:* At the start of the exercise session, following the warm-up, exercises in low positions are performed. These low positions relieve the back muscles from the mechanical stress of supporting the spine against gravity. It is likely that because of this, the largest correction of scoliosis is observed in these low bending positions. A “break” exercise is performed between exercises in low positions. A break exercise consists of maximum active kyphotization of the thoracic spine and lordotization of the lumbar spine (Fig. 66) with simultaneous 3D correction of the spine deformation.

**Fig. 66:**
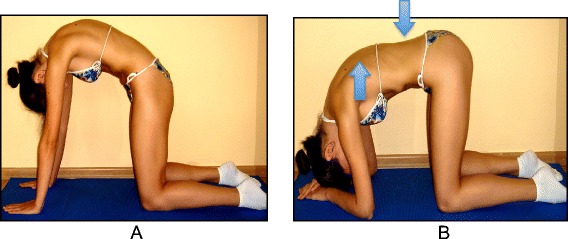
(**a**, **b**): Maximum active kyphotization of the thoracic spine (**a**) and lordotization of the lumbar spine (**b**) with simultaneous 3D correction of the spine deformation
3. *Active 3D auto-correction* in upright positions: Active 3D auto-correction exercises are performed in high positions (the spine is placed vertically) where the trunk muscles must work to support the spine against the full force of gravity (Figs. 67 and 68).

**Fig. 67:**
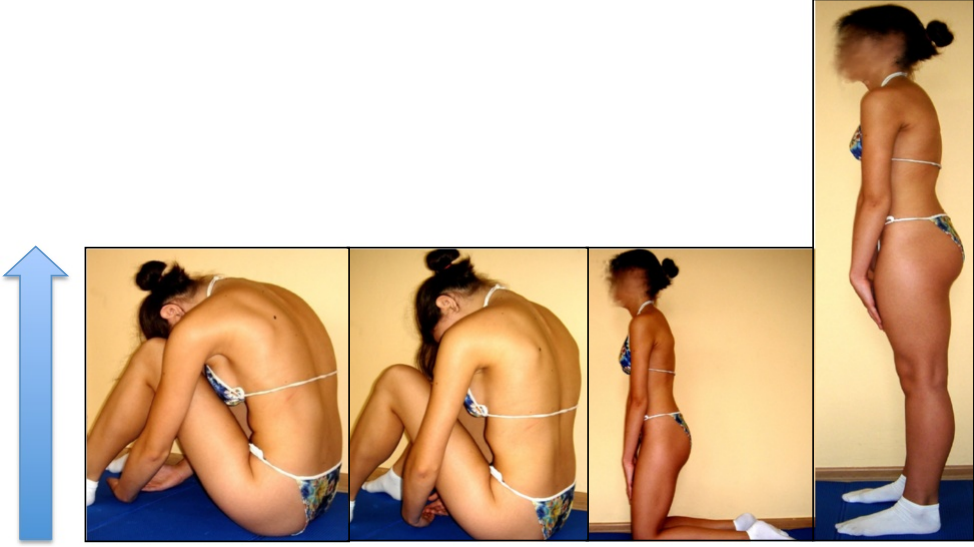
Photograph sequence of a patient achieving “high position” progression from sitting to kneeling and finally to standing, all while maintaining complete 3D correction of the spine deformation

**Fig. 68:**
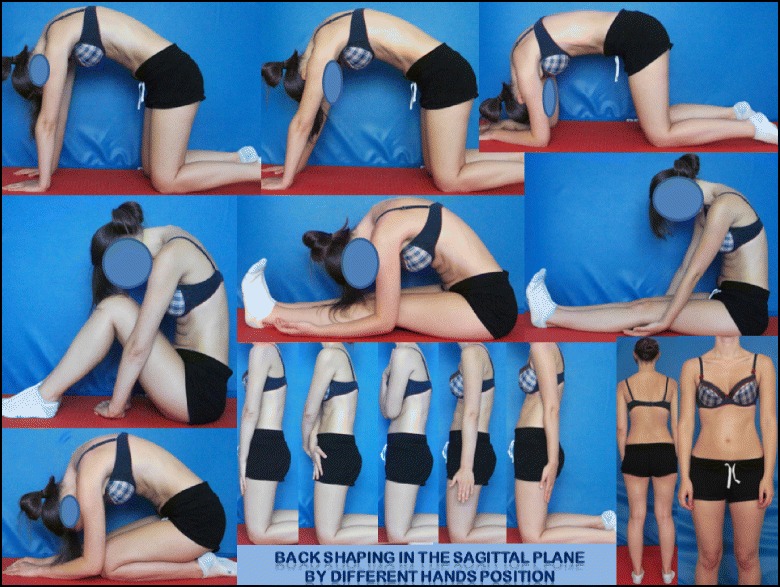
A summary of the different symmetrical positions of the DoboMed method

### Scientific evidence

Much research has been done on the Dobomed method of conservative scoliosis treatment. Short-term exercises in accordance with the Dobomed method bring about a significantly higher degree of improvement in respiratory function than other scoliosis treatment exercise methods [41] while at the same time improving trunk morphology values [42].

Dobomed physiotherapy can also be used to stabilize progressive thoracic scoliosis in patients wearing the Chêneau brace [37, 43]. Stabilization of scoliosis curves in children with the Dobomed method can also be seen radiographically [39, 44]. Asymmetric mobilization of the trunk in strictly symmetrical positions, as a part of the Dobomed method, has been shown to decrease Cobb angles and vertebral rotation [39], or, at minimum, halt scoliosis progression [43, 45, 46]. Moreover, asymmetrical mobilization significantly rebuilds physiological thoracic kyphosis in cases of idiopathic scoliosis accompanied by a straight back [44]. Additionally, conservative treatment of idiopathic scoliosis using the Dobomed method of intensive 3D respiratory exercises helps preserve normal exercise efficiency of patients with idiopathic scoliosis as measured by the ventilatory anaerobic threshold [41].

## Side shift (United Kingdom)

### Introduction

**Fig. 69:**
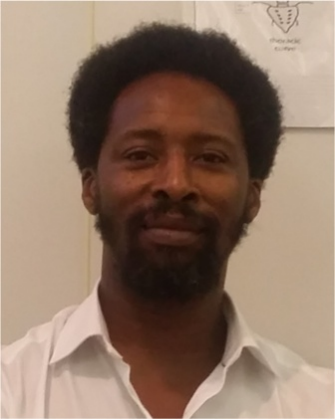
Tony Betts, school leader and physical therapist teaching the Side-Shift method (UK)

The Side Shift approach to conservative idiopathic scoliosis treatment, led by Tony Betts (Fig. 69), a physiotherapist who specializes in spinal deformities at the Royal National Orthopaedic Hospital in London, is built upon the theory that a flexible curve can be stabilized with lateral movements. Excessive side movements of the trunk correct the lateral deviation of the trunk along the coronal plane. These lateral movements promote a reduction in postural forces, which aim to affect the development of the structural curve.

### History

The Side Shift approach to correct scoliosis curves was developed by Dr. Min Mehta in 1984 and has been used by therapists at the Royal National Orthopaedic Hospital since that time. Dr. Mehta initially used this approach to treat congenital scoliosis curves in children; she proposed that growth could be a corrective force for spinal deformity in children [47]. Through repetitive actions the direction of growth opposes the curve of the spine. While correcting the curve position through trunk shifts, the body is using muscular forces and connective tissue stretches to increase mobility and re-align the soft tissue components of the scoliosis. It is believed that frequent repetition of corrective movements also helps to promote somatosensory integration of the spinal position to a more upright and physiological posture.

### Classification system

The consultants at the Royal National Orthopaedic Hospital use the King and Lenke systems for surgical classification of scoliosis curves. The Side Shift treatment method of scoliosis uses the King classification as a starting point for the descriptive classification of the curve types and diagnosis of the area of the curve. A classification system for Side Shift has been developed [48] which examines the mobility of the spine and its ability to correct towards the midline (schematic diagram X classification). According to this classification system, there are three types of scoliosis curves with classifications based on the flexibility of the curve and the ability of the patient to auto-correct the deformed spine during a side shift movement. *Type I* is any curve pattern that can be corrected by shifting the trunk beyond the coronal midline to the contralateral side of the scoliosis curve (very flexible curve). *Type II* is any curve pattern that can be corrected to the midline of the coronal plane, aligning the spine with the pelvis, with partial derotation of the vertebrae (moderately flexible curves). *Type III* is any curve pattern that cannot correct to the midline, remains shifted during the side shift exercise, and where the vertebrae do not de-rotate (extremely rigid curves, which may represent a severe structural curve).

### Treatment indications and goals

The Side Shift approach includes principles of the Schroth method and treatment indications as outlined in the 2011 SOSORT guidelines [9]. The goal of the Side Shift method of scoliosis treatment is the active correction of the spinal curve directed at the apex of the scoliosis, with Side Shift movements of the trunk towards the concavity, including active postural corrections in all planes. The starting point of analysis is the coronal plane (Cobb angle) curvature with emphasis towards the apex of the curve in this plane [49]. Core stabilization of the spine is also important through isometric lower abdominal, gluteal, and scapulae strengthening exercises that are all included in the treatment program. The main aim of the exercises for AIS is the correction of postural deviation from the midline in pre- or post-operative patients. In adults, the main aim is the reduction of mechanical pain caused by excessive postural deviations of the spine from the neutral midline. In adults, the sagittal plane is sometimes considered the important starting place for correction depending upon the radiological findings. Breathing exercises are included to improve expansion of the concave side of the curve and derotation of the ribs on the convex side of the curve, helping to improve vital capacity. Exercises to improve proprioception and balance with the emphasis on correcting posture and instructions to “trunk shift” during activities of daily living are added later into the treatment and show higher level of integration. A treatment pathway has been developed which guides the therapist in the treatment of AIS (Pathway 1).

### Age specifics

For adolescent scoliosis patients, exercises with overcorrection of the spine to the contralateral side of the scoliosis are recommended, but never to the point of pain. For adult scoliosis patients, exercises with correction to the physiological postural midline (neutral position) or pain-free positions are recommended. A separate adult pathway has also been developed to guide the safe treatment of adults with scoliosis. Pain management techniques are also included in the Side Shift method, including acupuncture, assisted bracing for support, medication and transcutaneous electrical nerve stimulation (TENS).

### 3D principles of correction

AIS Treatment Pathway:

In designing a treatment protocol the therapist has to answer these questions:

1. *What is the most important curve?* (for example, Cobb angle and the amount of rotation)
2. *Is shift/translation necessary?* (Measure: Rib overhanging – trunk frontal plumb-line)
3. *Is elongation necessary?* (Measure: plumb-line sagittal – slumped posture)
4. *Is derotation necessary?* (Measure: rib hump/ATR, breathing/twisting)
5. *Is lordosing/kyphosing necessary?* (Measure: sagittal plane alignment)
6. *What is the easiest movement to perform/remember?* (Only do corrective movements at home that are accurately/easily reproduced at clinic)

The Side Shift approach has been modified with practice, experience, and clinical re-evaluation. It includes several principles from the Schroth method, including active 3D auto-correction (along transverse, frontal, and sagittal planes), overcorrection movements beyond the midline, side shifting of the trunk in the direction opposite to the convexity of the primary curve, and repetition of corrective movements during growth to influence the direction of spinal growth. Moreover, the patient must be old enough to understand instructions and perform the exercises independently.

**Fig. 70:**
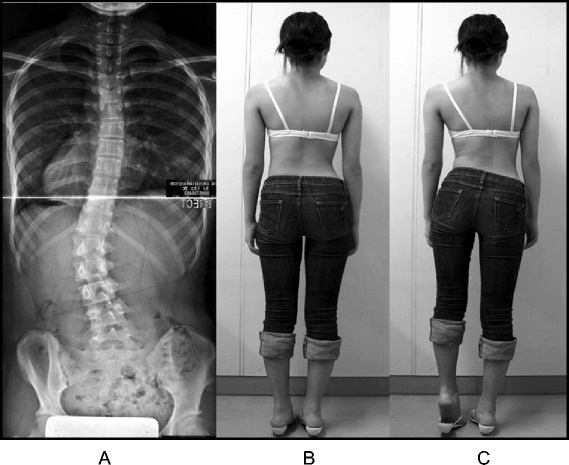
(**a**, **b**, **c**): “Hitch” exercise. A patient with a left thoracolumbar scoliosis curve seen on radiograph (**a**) stands in a neutral position (**b**). She is instructed to transition into the “hitch” position (**c**) by lifting her left heel on the same side as the convexity of the curve while keeping her hip and knee straight. The “hitch” position reduces the asymmetry of the patient’s waistline (**c**)

**Fig. 71:**
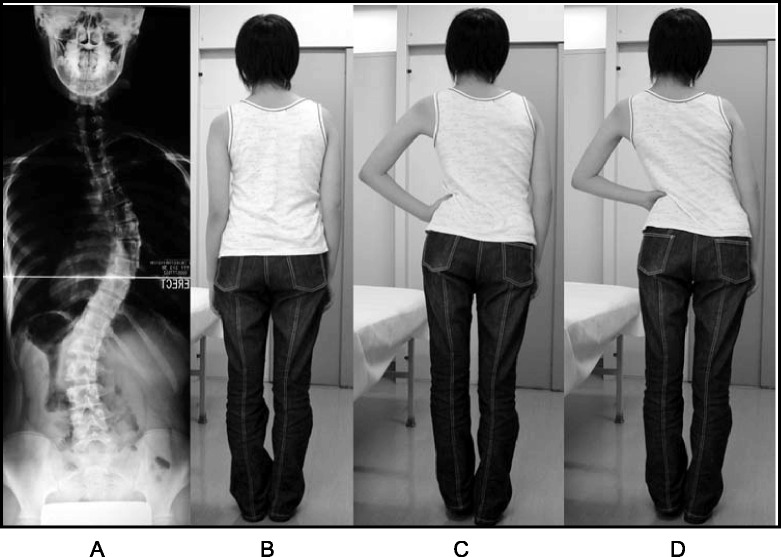
(**a**, **b**, **c**, **d**): The “Hitch-Shift” exercise is indicated for patients with double scoliosis curves. A patient with a double scoliosis curve seen on radiograph (**a**) stands in a neutral position (**b**). She is instructed to transition into the “hitch” position (**c**) by lifting her left heel on the same side as the convexity of the lumbar curve while keeping her hip and knee straight. She then immobilizes the lumbar curve using her hand and “shifts” her trunk to the concavity of the thoracic curve (**d**)

Two principle exercises of the Side Shift method include the hitch exercise and the hitch-shift exercise (Figs. 70 and 71). The hitch exercise is used for lumbar or thoracolumbar curves, whereas the hitch-shift exercise is an option for double major curves. During the hitch exercise, in the standing position, the patient is instructed to lift their heel on the convex side of the curve while keeping their hip and knee straight and to hold the “hitch position” for ten seconds before returning to a neutral position. The patient is required to repeat this exercise at least 30 times a day. In the hitch position, the pelvis on the convex side of the curve is lifted, resulting in a decreased lateral tilt at the inferior aspect of the vertebrae. This corrects the curve and reduces the asymmetry of the indented waistline. During the hitch-shift exercise, in the standing position, the patient is instructed to lift their heel on the convex side of the lower curve, as done in the hitch exercise, while immobilizing the lower curve with their hand and shifting their trunk to the concavity of the upper curve. The patient is required to hold this position for ten seconds before returning to a neutral position and to repeat this exercise at least 30 times a day.

### The use of breathing mechanics, muscle activation, and mobilization

**Fig. 72:**
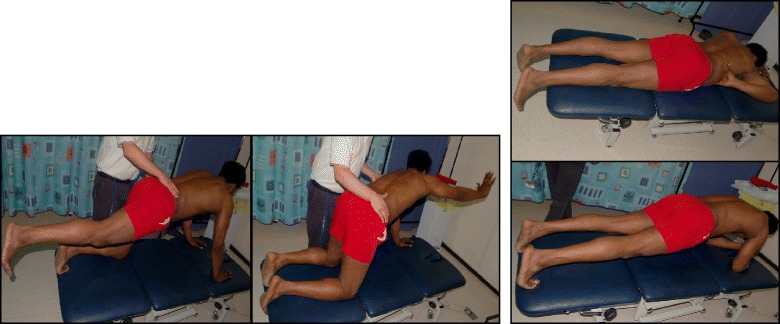
Trunk stabilization exercises using the side-shift method. The “bird-dog” (*left*) and the plank (*right*) exercises are performed while maintaining the side shift position

**Fig. 73:**
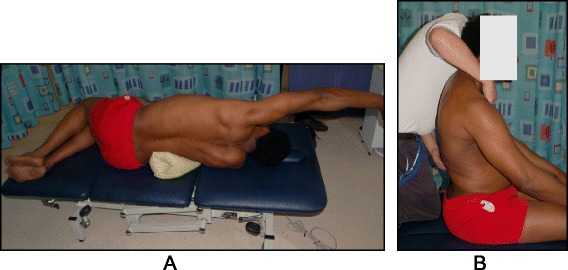
(**a**, **b**): Lateral (**a**) and sagittal (**b**) trunk mobilization exercises using the Side Shift method

The Side Shift approach uses the same breathing mechanics as the Schroth (Section 2) and DoboMed (Section 5) methods, based on the principles of rotational angular breathing that targets breathing into the concavity of the ribs. Muscle activation (Fig. 72) is achieved with isometric muscle bracing (via plank or ‘bird-dog’) to provide dynamic correction to the Side Shift corrective movement (incorporating Pilates and core strengthening exercises). Active muscle control helps to prevent muscle atrophy and provides greater forces to the corrective movements of the Side Shift method. The Side Shift method also includes the principles of Maitland and myofascial release techniques to increase mobilization and flexibility of the joint tissues and the soft tissues, respectively (Fig. 73).

### Treatment tools: active and passive

The Side Shift approach uses a combination of mirrors, photographs, and videos to assist patients with correction during exercises.

### Description of the most relevant exercise mechanics

**Fig. 74:**
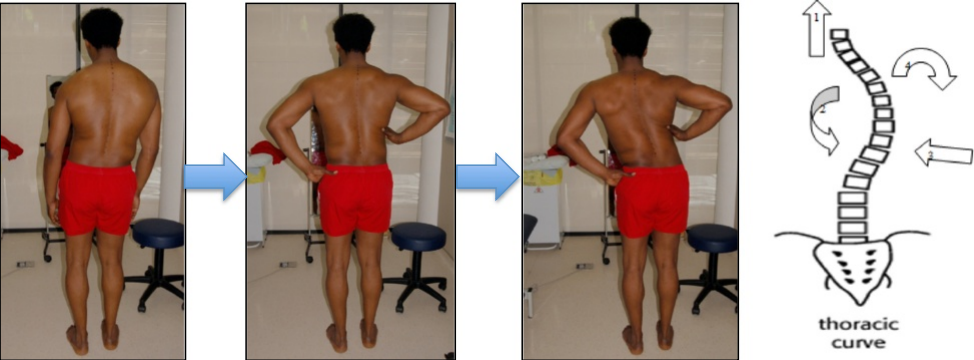
Patient with a right thoracic scoliosis curve demonstrates a sequence of Side Shift exercises with assistive correction in the standing position. The *arrows* in the diagram on the right illustrate the corrective movement of the spine during the Side Shift exercise

**Fig. 75:**
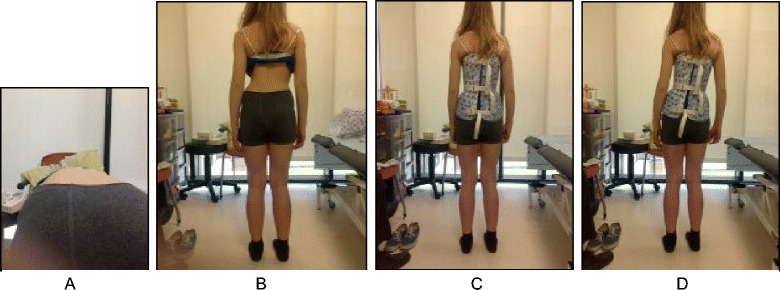
(**a**, **b**, **c**, **d**): Patient with a left thoracolumbar scoliosis curve (**a**, **b**) performs a sequence of side shift exercises to the right while wearing her brace (**c**, **d**)

**Fig. 76:**
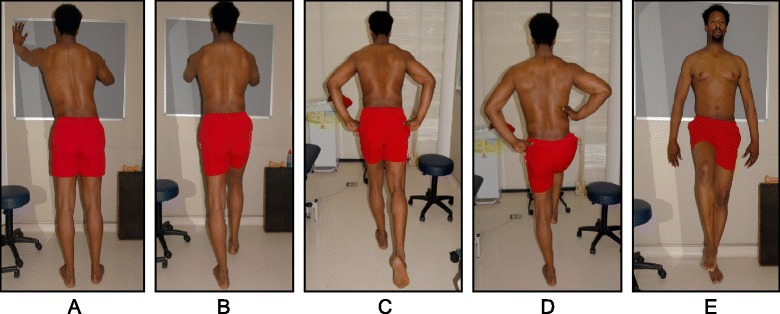
(**a**, **b**, **c**, **d**, **e**): Patient with a right thoracic scoliosis curve demonstrates side shift balance stabilization exercises against a wall (**a**, **b**) and in the standing position (**c**, **d**, **e**)

Side Shift exercises can be performed either in the sitting or standing position and either with or without a brace (Figs. 74 and 75). The patient actively “shifts” their trunk away from the convexity of the curve, while either sitting or standing, and holds the position for ten seconds. A more advanced exercise involves maintaining the side shift position away from the convexity of the curve while going from a seated to a standing position, and later on unsteady surfaces to challenge the balance and the proprioceptive system (Fig. 76). This exercise encourages transitional postural control during everyday movements.

### Activities of daily living and sport

**Fig. 77:**
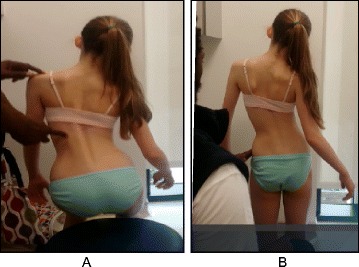
(**a**, **b**): Patient with a right thoracolumbar scoliosis curve performs a side shift to the left in a sitting-to-standing position (**a**) and in the standing position (**b**) as part of side shift exercises which can be done as part of activities of daily living (ADLs)

The Side Shift approach encourages the mantra of “think shift” with activities of daily living and incorporates side shift exercises into everyday movements, such as going from a sitting position to a standing position (Fig. 77).

### Scientific evidence

Dr. Min Mehta [47] first described the Side Shift exercise for the treatment of scoliosis in 1985. Dr. Mehta used single and multiple case reports of 35 patients with idiopathic scoliosis to demonstrate positive clinical and radiological corrections of scoliosis with Side Shift exercises. This study was part of an un-blinded retrospective study presenting observational and radiological results of over 2,530 patients. In 2002 and 2008 two studies from Japan [50, 51] that investigated the Side Shift method in 39 females with AIS concluded that Side Shift exercises and the hitch exercises are useful options for idiopathic scoliosis.

## Functional individual therapy of scoliosis (Poland)

### Introduction

**Fig. 78:**
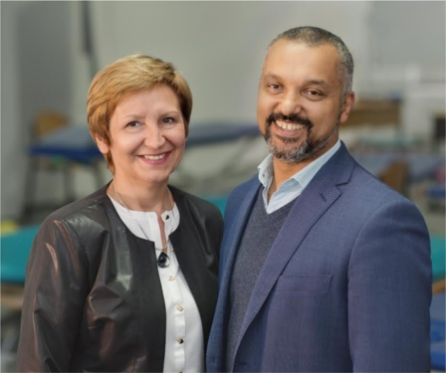
The Functional Individual Therapy of Scoliosis (FITS) school leaders, Marianna Białek and Andrzej M'hango

Functional Individual Therapy of Scoliosis (FITS) was created in 2004 by Marianna Białek PT, Ph.D., and Andrzej M'hango PT, M.Sc., D.O. (Fig. 78a-b). FITS treatment [52] is based upon the inclusion of many elements selected from a variety of other therapeutic approaches that have been adapted and modified to form a different scoliosis treatment concept. The FITS method also contains many techniques developed by the school leaders. FITS may be used as a separate system for scoliosis correction, as a supportive therapy for bracing, in preparation of children for surgery, or for the correction of the shoulder and pelvic girdles after surgical intervention [52]. The FITS method may also be applied to other structural and non-structural spinal deformities.

### History

Marianna Białek and Andrzej M'hango collaborated for the first time in the late 1990’s during a scoliosis manual therapy training course. At that time, both were involved in various courses, training sessions and conferences on the most recent physiotherapeutic methods for the conservative treatment of scoliosis. Between 1999 and 2000, they prepared and conducted the course “Proprioception Neuromuscular Facilitation (PNF) in Scoliosis” for physiotherapists. In 2002, they organized the first two-week rehabilitation camp for children with scoliosis. Taking advantage of their experience in treating young patients with scoliosis and the expertise of their colleagues, including Wieslaw Chwała of the Department of Biokinetics at AWF Kracow, who at the time was carrying out EMG examination and a 3D gait analysis in the Vicon system, they created their own program of scoliosis therapy. Through 2006, FITS (Functional Individual Therapy of Scoliosis) continued to evolve with the cooperation of Professor Tomasz Kotwicki from the Department of Pediatric Orthopedics and Traumatology, University of Medical Sciences in Poznań, Poland.

### Definition of treatment

FITS is a complex, asymmetrical and individual method of treating patients with scoliosis. It is based on a number of physiotherapeutic techniques from which the creators of the method selected those treatment approaches that they believed were most useful and then adapted them as necessary. In addition, many of the techniques used in FITS were developed by the creators of the method based on their own experience as physiotherapists [53]. A description of the FITS method, its treatment indications and guidelines was published by Białek, M’hango [52, 54] in 2008 and 2011.

FITS can be used in a child of any age regardless of the Cobb angle and has been proven to be significantly effective in a short period of time [55]. FITS requires a child to be active in the therapy process, which is guided by an experienced and specially trained therapist. FITS therapy may be conducted in an outpatient clinic or as a one to two-week in-patient treatment course. FITS encourages physiotherapists to work together with orthopedists and psychologists in treating the patient.

### Classification system

Unlike other methods of scoliosis treatment, the FITS method has no traditional classification system; rather, it relies on an individual approach. Each patient’s scoliosis is different with reference to the number of curves, the location of the curves, the degree of curvature and trunk rotation, the body’s compensation (structural or functional) for the deformity, the position of the scapulae and pelvis, the muscle tension, the setting of the sagittal plane, and the patient’s breathing patterns, sense of stability, coordination, and psychological state. Based on the individual characteristics of the patient’s scoliosis, the scoliosis deformity is described as low, moderate, or severe. Each patient is assigned an individualized treatment program based on their unique deformity.

### Treatment indication and guidelines

In line with other scoliosis treatment methods, the FITS indications for scoliosis treatment are based on the 2011 SOSORT guidelines [9] with some modifications. Observation is not recommended for JIS patients. Rather, it is recommended that all JIS patients receive FITS therapy regardless of age or Cobb angle. If a JIS patient requires bracing, only rigid bracing is recommended. Soft bracing is not part of the FITS indication for therapy in any age group. FITS recommends part-time rigid bracing for JIS curves 21°-25° degrees and full-time rigid bracing for JIS curves greater than 26°. For AIS patients, observation only is recommended in curves up to 15°. Curves greater than 15° require FITS therapy regardless of the Cobb angle. For AIS curves greater than 30° in patients with Risser 0–2 (bone maturity), full-time rigid bracing is recommended. Additionally, patients can participate in FITS’s Special Inpatient Rehabilitation program regardless of Cobb angle, Risser sign, and age.

### Treatment goals and age specifics

FITS goals are divided into short-term goals and long-term goals. The short-term goals of FITS include increased patient awareness (psychological goal), improved shoulder and pelvic girdle alignment (aesthetic goal), patient education of 3D breathing and improved function, myofascial release, and teaching the correct shift. The long-term goals of FITS include decreasing scoliosis, stabilizing the scoliosis (stop curve progression), and improving aesthetics and body function in patients who do not undergo surgery or who are post-operative patients.

The nine main goals of the FITS concept:

1. Awareness of existing deformation of the spine and the trunk, and of the direction of the scoliosis correction.
2. Sensory-motor balance training (Fig. 79).

**Fig. 79:**
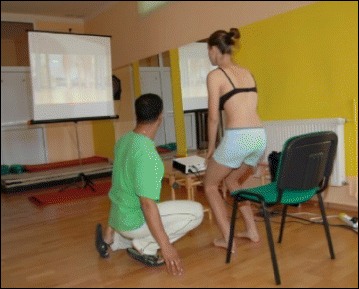
FITS method sensory-motor balance training. With a video camera positioned behind her, the patient is able to see her posture on the screen in front of her in real-time while moving from the sitting to the standing position and make corrections according to the instructions of the physical therapist
3. Release of myofascial structures that limit three-plane corrective movement (Fig. 80).

**Fig. 80:**
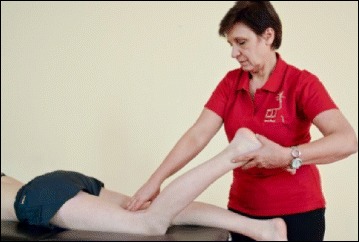
Physical therapist demonstrating the release of myofascial structures (relaxation of the hamstring muscles) that limit three-plane corrective movements
4. Lumbo-pelvic stabilization (Fig. 81).

**Fig. 81:**
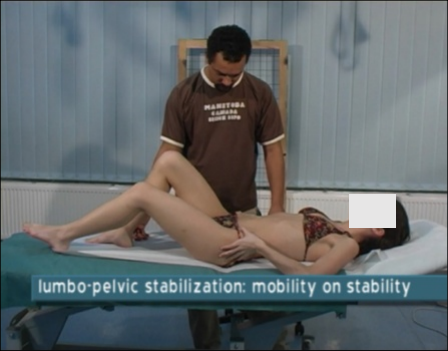
Lumbo-pelvic stabilization. The physical therapist instructs the patient to slowly extend her left hip while maintaining a stable lumbar spine and pelvis with the support of her left hand
5. Correction shift of the spine in frontal plane in order to correct the primary curve while stabilizing (or maintaining in correction) the secondary curve (Fig. 82).

**Fig. 82:**
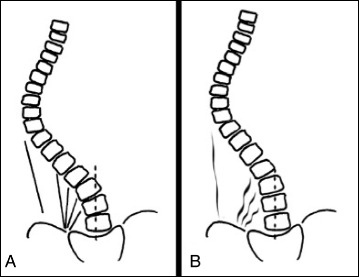
Correction shift in the frontal plane is more difficult when the myofascial structures limiting the corrective shift. Diagram shows the before therapy (**a**), and after myofascial therapy (**b**)
6. Facilitation of three-plane corrective breathing in functional positions and brace wearing (Figs. 83, 84, 85).

**Fig. 83:**
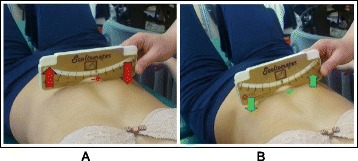
(**a**, **b**): Corrective breathing exercises in the supine position with a scoliometer, which shows the patient and the physical therapist where the rib expansion is occurring during expiration (**a**) and inspiration (**b**)

**Fig. 84:**
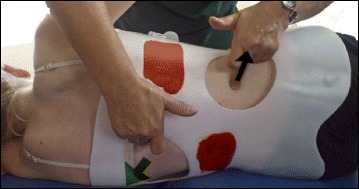
In-brace 3D corrective breathing. The patient is instructed to breathe into the concavities while wearing her Chêneau-style brace. The *arrows* show the direction of breathing and the *red markings* on the patient’s brace showing the convexities

**Fig. 85:**
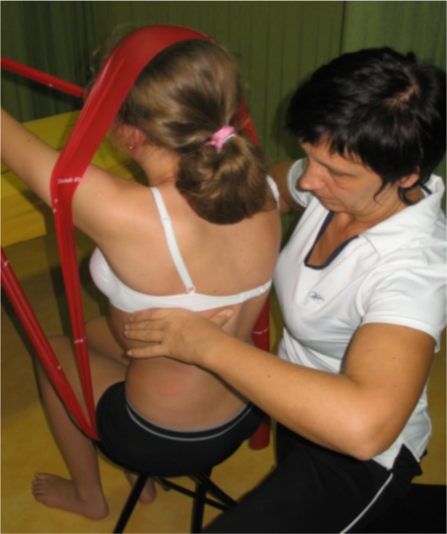
A physical therapist assists a patient with 3D correctional breathing in the sitting position, while a red elastic band facilitates auto-elongation and auto-correction of the scoliosis curves at the same time as providing resistance against the corrective movements
7. Correction patterns of scoliosis. Postural re-education.
8. Auto-correction in activities of daily living.
9. Clinical improvement, decrease or stabilization of the scoliosis curvatures.

FITS employs the same treatment protocol for JIS and AIS. For adult and elderly scoliosis patients FITS uses different protocols.

Juvenile idiopathic scoliosis (JIS) defines patients from 3 to 10 years old. For patients from 3 to 5 years old, FITS focuses on sensory motor balance exercises and lower trunk stabilization. Correction patterns are also used for children with scoliosis. Most of these exercises are carried out in the form of a game.

For children 6 to 10 years old, the full FITS method is used. FITS also focuses on sensory motor balance exercises and lower trunk stabilization.

For adolescent idiopathic scoliosis (AIS), 11 to 18 years old, FITS applies principles intended to prevent curve progression before the end of the patient's growth process.

For adults over the age of 18, the main goals are to improve the clinical view, eliminate pain, and prevent scoliosis progression.

### 3D Principles of correction: The 3 Stages

The FITS concept consists of three main stages of correction: patient examination and education, preparation for correction, and three-plane correction.

#### Stage I

**Fig. 86:**
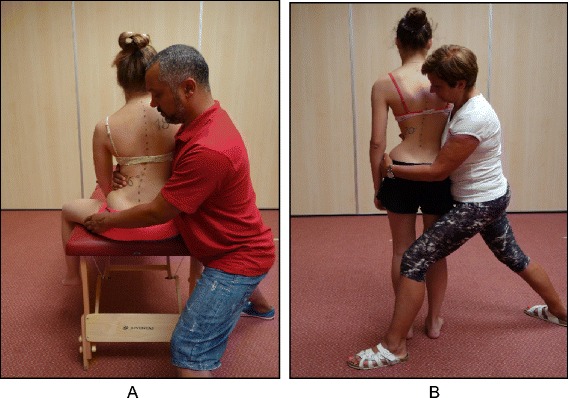
(**a**, **b**): A physical therapist demonstrates how to test the flexibility of the scoliotic spine in the sitting (**a**) and standing (**b**) positions

**Fig. 87:**
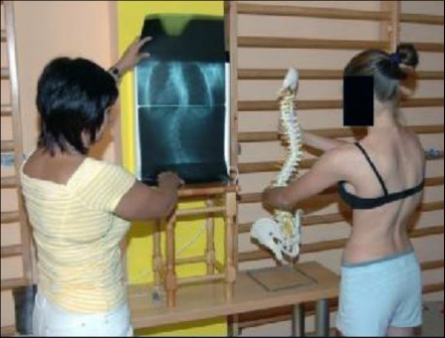
Physical therapist using radiographs and spine models to help the patient visualize and gain awareness of her trunk deformity caused by scoliosis

Patient examination and education include physical examination of the flexibility of the scoliotic spine in functional positions and patient education to help the patient become aware of their deformity (Figs. 86 and 87).

#### Stage II

**Fig. 88:**
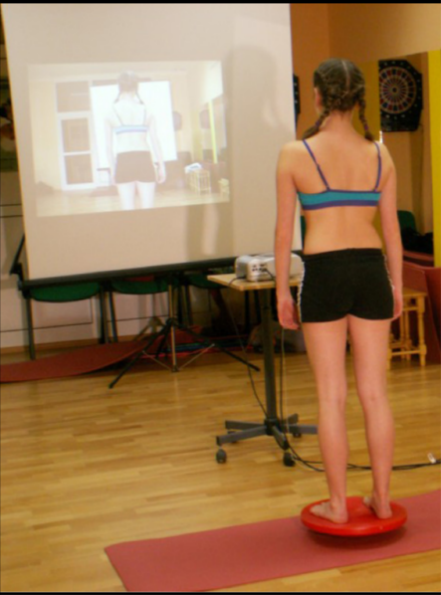
FITS sensory-motor balance training. With a video camera positioned behind her, the patient is able to see her posture on the screen in front of her in real-time while making postural corrections on the balance board

**Fig. 89:**
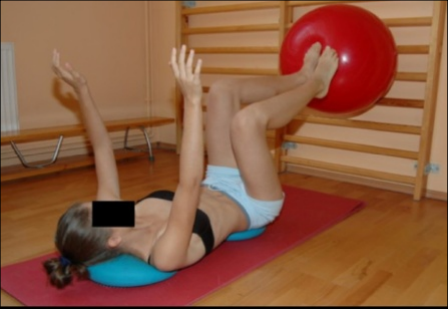
FITS sensory-motor balance training. The patient lies supine on two blue discs and tries to achieve perfect balance while holding up a Swiss ball against a wall bar with her feet

**Fig. 90:**
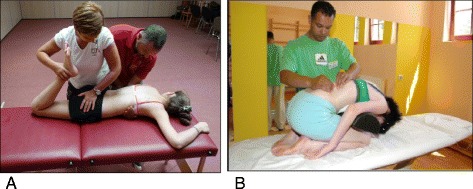
(**a**, **b**): **a** One physical therapist performing active relaxation of the rectus femoris while a second physical therapist uses a scoliosis derotation maneuver. **b** Myofascial release of the erector spinae muscles by a physical therapist while the patient bends forward over a foam roll

Preparation for correction: sensory motor balance training, detection and elimination of myofascial tension, which restricts three-plane corrective movements (Figs. 88, 89 and 90).

#### Stage III

3D correction, building, and stabilization of corrective patterns in functional positions.

**Fig. 91:**
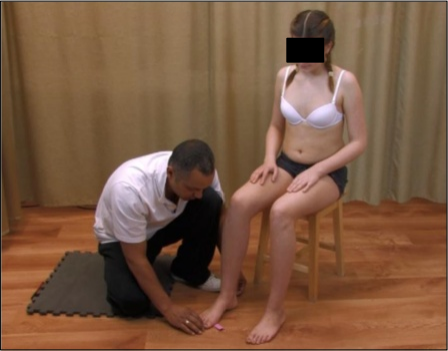
Corrective foot loading. A physical therapist teaches the patient correct weight bearing on the feet in the sitting position with stabilization of corrective

**Fig. 92:**
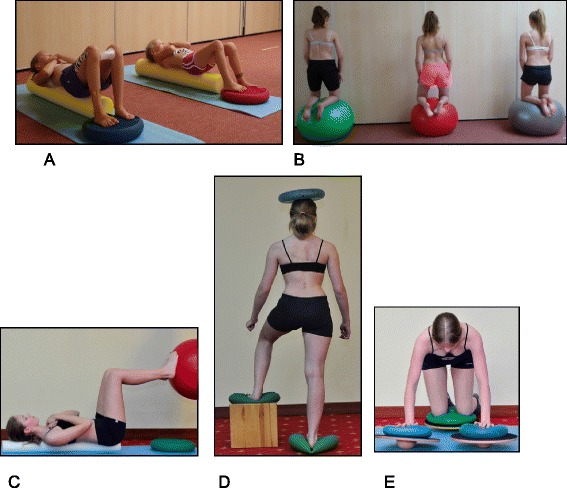
(**a**, **b**, **c**, **d**, **e**): FITS stabilization exercises. **a** Patients lying supine on a foam roll with their feet on a balance disc. **b** Patients kneeling on a Swiss ball while maintaining postural correction and balance. **c** Patient lying supine on a foam roll tries to maintain postural stability while holding up a Swiss ball against a wall bar with her feet. **d** A patient standing on two balance discs while balancing another disc on her head works on curve-specific postural stabilization. **e** An advanced FITS stabilization exercise where the patient balances on her hands and knees on balance discs atop balance boards

**Fig. 93:**
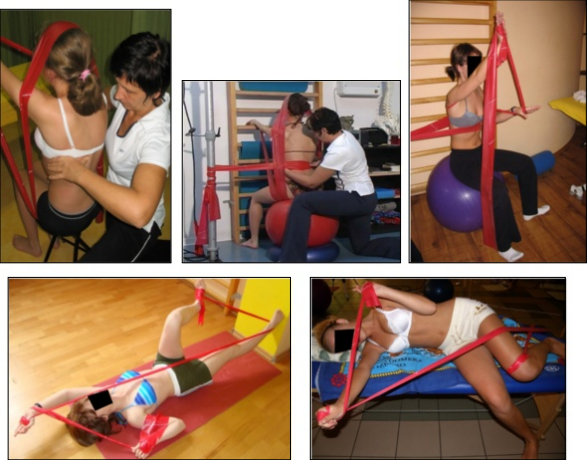
Patients demonstrating typical FITS exercises with elastic bands, which help facilitate the scoliosis specific correctional patterns. Initially, exercises are performed with the assistance of a physical therapist and then later are performed independently

These begin with correct foot loading (Fig. 91) and stabilization exercises (Fig. 92a–e ), then move on to performing corrective patterns (Fig. 93).

### Description of the most relevant exercise mechanics

The most relevant exercise mechanics of FITS are as follows:

1. Sensory motor balance training to improve nervous system control over the muscle’s function.
2. Mobilization and myofascial techniques to eliminate myofascial restrictions which limit three-plane corrective movement.
3. 3D correction breathing to increase derotation movement and improve breathing mechanism.
4. Corrective patterns (Fig. 94) – active correction.

**Fig. 94:**
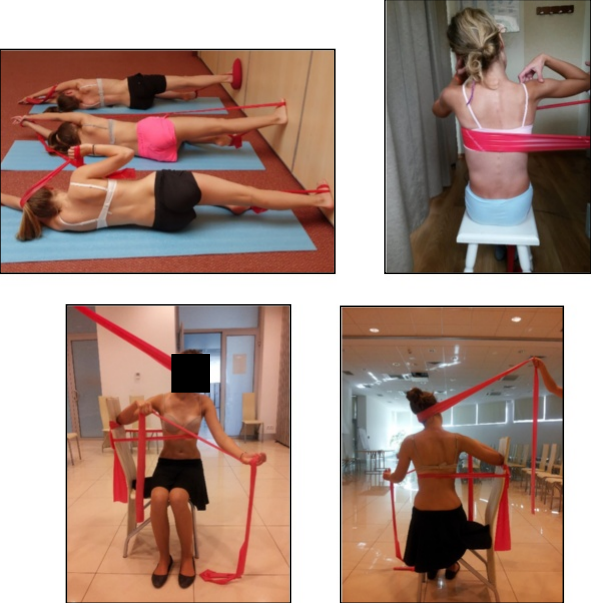
Patients demonstrating typical FITS exercises with elastic bands, which help facilitate the scoliosis specific correctional patterns
5. Neuromuscular re-education.
6. Auto-correction in activities of daily living.

### The use of breathing mechanics, muscle activation, and mobilization

FITS promotes breathing into the concavities of the ribcage. Using a scoliometer in supine position, the degree of correction with breathing exercises can be assessed. FITS promotes a gradual progression to the point where the patient performs breathing exercises in a functional position (sitting and standing). Muscle activation creates corrective tension in the trunk, thereby assisting in pelvic and spinal stabilization in the corrective pattern. Myofascial release is important to promote mobilization and flexibility of the spine before correction.

### Treatment tools: active and passive

Initially the physiotherapist assists the patient with manual correction using tools that provide biofeedback for the patient, such as video cameras, TV screens, and mirrors. Using rolls, sensorimotor pillows, balls, and balance trainers helps the patient improve proprioception. As the patient develops muscle memory through motor learning and sensorimotor feedback, the patient is able to make auto-corrections by themselves without the need for treatment tools.

### Activities of daily living and sport

Integration between correction and activities of daily living involves three training stages:

1. Auto-correction in sitting position: while maintaining the correction, the patient attempts to perform various ADLs such as brushing hair, putting on/taking off a shirt, putting on/taking off socks, and going from a standing to a sitting position (Fig. 95).

**Fig. 95:**
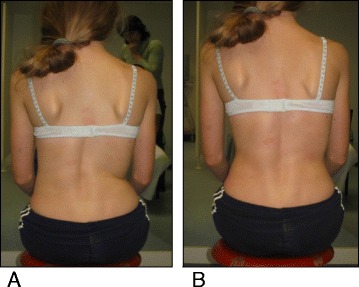
(**a**, **b**): Posture correction in the sitting position, before correction (**a**) and after active self-correction (**b**)
2. Auto-correction in standing position: while maintaining the correction, the patient attempts to perform the same activities of daily living as in Stage 1.
3. Auto-correction in walking.

### Scientific evidence

Since the creation of the FITS method in 2004, there have been numerous studies in co-operation with other specialists (physiotherapists, orthopedists, biomechanics, pulmonologists) on the effectiveness of this method in children with scoliosis. These include studies of the impact of the FITS method on change of Cobb angle [53], angle of trunk rotation and scoliotic posture [55–58].

In 2011, Bialek [53] conducted a study analyzing the effect of the FITS method and bracing on (1) single or (2) double structural scoliosis with Cobb angle 10–25° (Group A) and on patients with Cobb between 20–45° (Group B). In Group A: (1) in single scoliosis, 50.0 % of patients improved, 46.2 % were stable and 3.8 % progressed, while (2) in double scoliosis, 50.0 % of patients improved, 30.8 % were stable and 19.2 % progressed. In group B: (1) in single scoliosis, 20.0 % of patients improved, 80.0 % were stable, and no patient progressed, while (2) in double scoliosis, 28.1 % of patients improved, 46.9 % were stable and 25.0 % progressed. The study concluded that best results were obtained in 10–25° scoliosis, indicating that therapy should begin where a patient shows a 10–25° curve in order to prevent greater structural changes within the spine.

The study of the effectiveness of FITS Therapy in Early Onset Idiopathic Scoliosis (range 4 to 9 years old) was published in Medicine [59]. In this study a significant decrease of Cobb angle and angle of trunk rotation was observed in close to 80 % of the subjects.

**Fig. 96:**
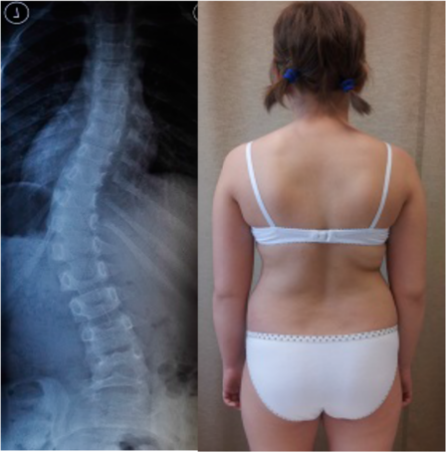
A 12-year-old female with a *left* thoracolumbar scoliosis has an improved physical appearance and aesthetic after being treated with bracing and the FITS method

**Fig. 97:**
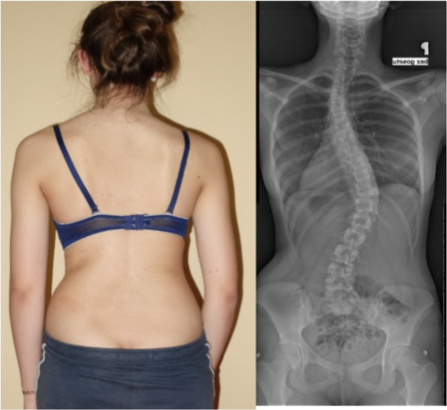
A 15-year-old female patient with a 32° Cobb angle *left upper* thoracic curve and a 36° Cobb angle right thoracolumbar curve as seen on the radiograph on the *right*, has an improved physical appearance and aesthetic with FITS therapy

Regardless of the angle of curvature, studies demonstrate that FITS therapy has a beneficial effect on the clinical appearance of each child (Fig. 96 and 97).

## Conclusion

Seven major schools operating under the SOSORT banner focus on the treatment of adolescent idiopathic scoliosis. As can be seen by the therapeutic approach of each school, and the scientific evidence supporting each school’s method of treatment, each of the seven schools promotes a unique technique and unique exercises. However, the schools’ overall goals are the same, as each method seeks to treat all aspects of the 3D scoliosis deformity by realigning the spine, rib cage, shoulders and pelvis to ‘normal’ anatomical postures (see Table 1). The evidence supporting the effectiveness of PSSE is growing, with more high quality research studies being published in recent years. The research must continue in order to further study the effectiveness of the various schools and to determine which methods and which exercises are most beneficial for patients.

**Table 1:** The similarities and the differences between the methods

School name	Treatment indication	Age treated	Principles of correction	Classification system	Breathing technique	Mobilization and flexibility	Brace used	Evidence
Lyon (France)	SOSORT 2011 guidelines	* Juvenile Adolescent ** Adult	Focuses on physical therapy exercises in preparation for brace wearing and in brace	Ponseti Lenke	Rotational angular breathing (RAB)	Encouraging thoracic kyphosis and lumbar lordosis	3D ARTbrace (Asymmetrical Rigid Torsion brace)	De Mauroy JC et al. Scoliosis. 2015;10:26
Schroth (Germany)	SOSORT 2011 guidelines	* Early onset * Juvenile Adolescent ** Adult	Reshape the thorax through isometric muscle activation around the prominences (the convexities) and a specific breathing technique (called rotational angular breathing (RAB) or simply orthopedic breathing) in the collapsed areas (the concavities)	Katharina Schroth’s Body Blocks	Rotational angular breathing (RAB)	Rib cage, spine mobilization and lower extremities flexibility	3D Chêneau brace	RCT - Schreiber S et al. Scoliosis. 2015;10:24 Weiss H et al. Hard Tissue. 2013; 2(3): 27 RCT - Kuru T et al. Clinil Rehabil. 2015 Weiss HR et al. Stud Health Technol Inform. 2002;91:342–7
Scientific Exercise Approach to Scoliosis (SES) (Italy)	SOSORT 2011 guidelines	Same treatment objectives for all ages	Educates and trains patients to actively self-correct their posture and to incorporate that self-correction into functional exercises	Ponseti	Rotational angular breathing (RAB)	Pre-bracing mobilization	3D Sibilla brace (Cobb <30°) Sforzesco brace (Cobb 30°–50°)	RCT - Monticone M et al. Eur Spine J. 2014;23(6):1204–14 Negrini S et al. *Disability and Rehabilitation* 2008;30(10):772–785
Barcelona Scoliosis Physical Therapy School (BSPTS) (Spain)	SOSORT 2011 guidelines	* Juvenile Adolescent ** Adult	Based on the original principles of correction established by Katharina Schroth. Like the Schroth method, the school’s aim to improve the scoliotic posture via muscle activation and the RAB technique mentioned above.	Katharina Schroth’s Body Blocks and Manuel Rigo’s radiological classification	Rotational angular breathing (RAB)	Rib cage, spine and lower extremities flexibility	3D Rigo Chêneau brace	Schroth evidence above plus: Rigo M et al. *Scoliosis* 2010,5:1 Otman SN et al. Saudi Med J. 2005; 26(9): 1429–35
Dobomed (Poland)	SOSORT 2011 guidelines	Same treatment objectives for all ages	Involves mobilization of the primary curve toward curve correction with a special emphasis on ‘kyphotization’ of the thoracic spine and/or ‘lordotization’ of the lumbar spine in closed kinetic chains	Dobomed	Specific Rotational angular breathing in a ‘phased-lock’ respiration technique	Increase thoracic spine kyphosis and lumbar lordosis	3D Cheneau brace	Durmala J et al. *Ortop Traumatol Rehabil*. 2003;5(l):80–5 Durmala J et al. *Scoliosis*. 2009;4(Suppl 2):029
Side-Shift (United Kingdom)	SOSORT 2011 guidelines	Adolescent ** Adult	Built on the theory that a flexible curve can be stabilized with lateral movements and that repetitive side movements of the trunk will correct the lateral deviation of the trunk along with the coronal plane	King	Rotational angular breathing (RAB)	Principles of Maitland and myofascial release techniques	Unspecified	
Functional Individual Therapy of Scoliosis (FITS) (Poland)	SOSORT 2011 guidelines	Juvenile Adolescent ** Adult	Based on a number of physiotherapeutic techniques that were selected and adapted specifically for the treatment of scoliosis. Examples are Proprioception Neuromuscular Facilitation (PNF) and myofascial release techniques	No traditional classification system	3D corrective breathing into the concavities	Mobilization and myofascial techniques to eliminate myofascial restrictions	3D Cheneau brace	Białek M. *Scoliosis*. 2011;28;6(1):25 Białek M. *Medicine*. 2015;94(20):e863

* Exercises are modified to allow participation of young children

** Exercises are modified and focus on reduction of pain

## Abbreviations

ADL, activity of daily living; AIS, adolescent idiopathic scoliosis; ARTbrace, Asymmetrical Rigid Torsion brace; BSPTS, Barcelona Scoliosis Physical Therapy School; CSL, central sacral line; CSN, Centro Scoliosi Negrini; EOS, early onset; FITS, Functional Individual Therapy of Scoliosis; ISICO, Instituto Scientifico Italiani Colonna Vertebrale; JIS, juvenile idiopathic scoliosis; LEV, lower end vertebra; N3N4, Non 3-Non 4 scoliosis pattern; PNF, proprioception neuromuscular facilitation; PSSE, Physiotherapy Scoliosis Specific Exercises; QOL, quality of life; RAB, rotation angular breathing; RCT, randomized controlled trial; SCT, shoulder counter-traction; SEAS, Scientific Exercises Approach to Scoliosis; SOSORT, The Society of Scoliosis Orthopaedic Rehabilitation and Treatment; SRS, Scoliosis Research Society; SRS-22r, Scoliosis Research Society 22r questionnaire; ST, shoulder traction; TENS, transcutaneous electrical nerve stimulation; TL, thoracolumbar; UEV, upper end vertebra; 3D, three-dimensional; 3C, three-curve scoliosis pattern; 4C, four-curve scoliosis pattern
